# Engineering zero modes in transformable mechanical metamaterials

**DOI:** 10.1038/s41467-023-36975-2

**Published:** 2023-03-07

**Authors:** Zhou Hu, Zhibo Wei, Kun Wang, Yan Chen, Rui Zhu, Guoliang Huang, Gengkai Hu

**Affiliations:** 1 School of Aerospace Engineering, Beijing Institute of Technology, Beijing, 100081 China; 2 School of Mechanical Engineering, Tianjin University, Tianjin, 300350 China; 3 Key Laboratory of Mechanism Theory and Equipment Design of Ministry of Education, Tianjin University, Tianjin, 300350 China; 4 Beijing Institute of Technology Chongqing Innovation Center, Chongqing, 401120 China; 5 Department of Mechanical and Aerospace Engineering, University of Missouri, Columbia, MO 65211 USA

**Keywords:** Mechanical engineering, Mechanical properties

## Abstract

In the field of flexible metamaterial design, harnessing zero modes plays a key part in enabling reconfigurable elastic properties of the metamaterial with unconventional characteristics. However, only quantitative enhancement of certain properties succeeds in most cases rather than qualitative transformation of the metamaterials’ states or/and functionalities, due to the lack of systematic designs on the corresponding zero modes. Here, we propose a 3D metamaterial with engineered zero modes, and experimentally demonstrate its transformable static and dynamic properties. All seven types of extremal metamaterials ranging from null-mode (solid state) to hexa-mode (near-gaseous state) are reported to be reversibly transformed from one state to another, which is verified by the 3D-printed Thermoplastic Polyurethanes prototypes. Tunable wave manipulations are further investigated in 1D-, 2D- and 3D-systems. Our work sheds lights on the design of flexible mechanical metamaterials, which can be potentially extended from the mechanical to the electro-magnetite, the thermal or other types.

## Abstract (web summary)

Reconfigurable elastic properties are essential for functional mechanical metamaterials. Here, the authors propose a 3D metamaterial with engineered zero modes, and experimentally demonstrate its transformability covering all seven extremal metamaterial types, leading to programmable wave functions.

## Introduction

Mechanical metamaterials^1^ are a type of architected materials whose rationally designed microstructures allow them to have counterintuitive properties that are unavailable among the conventional materials, e.g., negative Poisson’s ratio^2–4^, graded stiffness^5^, non-reciprocal response^6^, etc.^7,8^. Most recently, the development of two-dimensional (2D) or three-dimensional (3D) metamaterials^9,10^, after the acquisition of unconventional physical properties and programmability, has entered the stage of tunability^11–19^, an ability for the metamaterial’s properties to be tuned through deformation controlled by the embedded actuation or external stimuli, which leads to the flexible metamaterial^20^. Traditional origami structure and modular origami structure^21–23^ provide a powerful platform for designing 3D flexible metamaterials not only with multiple deformation paths^24^ but also tunable material properties^25–32^ such as tunable negative Poisson’s ratio^33^.

No matter the targated properties of flexible metamaterials, their large deformation in most of cases is due to the elastic joints between the origami panels or modulars^25,26,34–36^. Such flexible connectivity makes the structural material exhibit a number of zero modes, i.e., deformation modes that cost little to no elastic energy^37^. While the aforementioned properties utilize the zero modes for the large deformation aiming at tuneability, one of the most spectacular and direct applications of zero modes is in the design of acoustic “invisibility” cloaks by identifying penta-mode materials^38–41^. In ideal elasticity, the number of zero modes in the 3D structures can alter the ordinary elasticity tensor from the state of null-mode (solid-state) to the states of uni-mode, bi-mode, tri-mode, quadra-mode, penta-mode and the state of hexa-mode (near-gaseous state), which bring extremal static as well as dynamic properties^38,42,43^. Recently, the polar metamaterial with uni-mode was suggested for perfect mechanical cloaks^44,45^. A 3D quadra-mode metamaterial was designed and an out-of-plane shear (SH) wave polarizer was proposed based on the metamaterial^43^. However, due to the lack of a blueprint for the systematic designs of the corresponding zero modes, little to no work has succeeded in realizing those materials with the specific number of zero modes or the capability to be transformed among all seven different zero modes.

In this paper, inspired by the modular origami design, we propose a type of transformable mechanical metamaterials by shaping joint linkages, which can be reconfigured among null-, uni-, bi-, tri-, quadra-, penta-, and hexa-modes, leading to tunable mechanical properties and reprogrammable wave functionalities.

## Results

### Decoupled orthogonal design of 3D transformable metamaterial unit and its tessellation

**Fig. 1:**
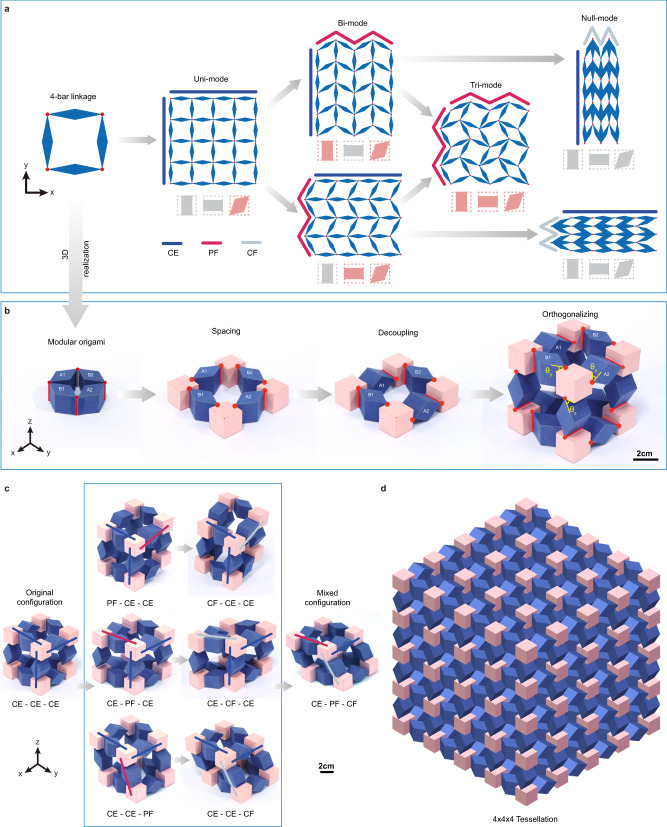
Design concept and 3D tessellation for transformable mechanical metamaterial. **a** 2D transformable tessellation consisting of periodically arranged equilateral 4-bar linkages, whose typical configurations include completely folded (CF), completely extended (CE) and partially folded (PF) configurations, and are further interpreted as continuous media with different numbers of zero modes (pink colored). **b** 3D realization of the transformable metamaterial via modular origami technique. **c** The decoupled motion in three orthogonal planes. **d** A 4 × 4 × 4 tessellation of 3D transformable metamaterial.

In the topological design of structure, a real revolute joint or weakened connection, also called compliant joint, permits zero mode under certain load type. Starting with an equilateral 4-bar linkage with four diamond bars and four revolute joints, we can construct a transformable 2D tessellation whose permitted zero modes are determined by different configurations of the 4-bar linkages (Fig. 1a), and we can interpret and categorize these states of the 2D tessellation via a homogenization method under Cauchy-Born hypothesis (Supplementary Note 1). From a continuous medium’s point of view, the 2D effective elasticity tensor has three eigenvalues, and based on the number of zero eigenvalues (0, 1, 2 or 3), the following four media can be categorized, namely null-, uni-, bi- and tri-mode materials. First, when all the 4-bar linkages in the 2D tessellation are completely extended (CE), the links in both x and y directions are collinear, hence, it can hold pure axial loading along the links, but becomes shearless, which indicates that the 2D tessellation is a uni-mode. Next, when the links in x or y direction are partially folded (PF), the 2D tessellation cannot withstand the axial loading in the corresponding PF direction, which makes it a bi-mode. Certainly, when the links in both directions are PF, the 2D tessellation turns into a tri-mode, which cannot withstand any loadings. Finally, when the links in x or y direction are completely folded (CF), the 2D tessellation becomes a null-mode, which can withstand all axial and shear loadings in the 2D space. Therefore, a transformable 2D metamaterial can be engineered based on the proposed 2D tessellation. The metamaterial behaviors like a null-mode, a uni-mode, a bi-mode or a tri-mode extremal material when the 2D tessellation is reconfigured in the CF, CE, PF-in-one-direction and PF-in-two-direction configurations, respectively (see Supplementary Note 2 and Supplementary Movie 1).

Although all possible 2D and 3D extremal materials were theoretically conceived by Milton and Cherkaev in 1995^38^, there is still an omnipresent challenge needs to be answered: can we develop a design strategy to physically engineer a 3D zero-mode metamaterial, which can freely transform elasticity tensors among all seven types of 3D extremal materials covering the solid null-mode to the gas-like hexa-mode? The key to answer the question is the realization of the decoupled 4-bar linkage motions in the three orthogonal planes in the 3D system. First, the modular origami technique is introduced to build a 3D linkage (Fig. 1b). By adding four pink cubes into the 3D linkage, the required connecting spaces are provided for the following orthogonal design. However, with all compliant joints (marked in red short lines) being arranged along the z-axis and forming a 2D closed loop, an 8-bar linkage forms instead, which confines all motions within the x-y plane. To break the in-plane confinement, we rotate the two rhomboids (A1 and A2) and arrange their joints along the y-axis. Hence, a 4-bar linkage supporting decoupled in-plane and out-of-plane motions is formed. Next, the same design strategy is applied to the other two orthogonal planes, and then a cubic unit is constructed. Thanks to the decoupled orthogonal design, switch among the three basic configurations (CE, CF and PF) can be performed along the three orthogonal directions independently (Supplementary Movie 2), and the desired 3D unit configuration can therefore be achieved through a superposition of the basic configurations (Fig. 1c). In fact, all possible configurations of the cubic unit can fulfill a 3D workspace (Supplementary Note 3). Finally, by using the cubic symmetry of the unit, we can easily tessellate it along the three orthogonal directions to construct a 3D metamaterial. Figure 1d shows a 4×4×4 tessellation built by the modular origami technique.

### Experimental characterization of the transformable metamaterial

**Fig. 2:**
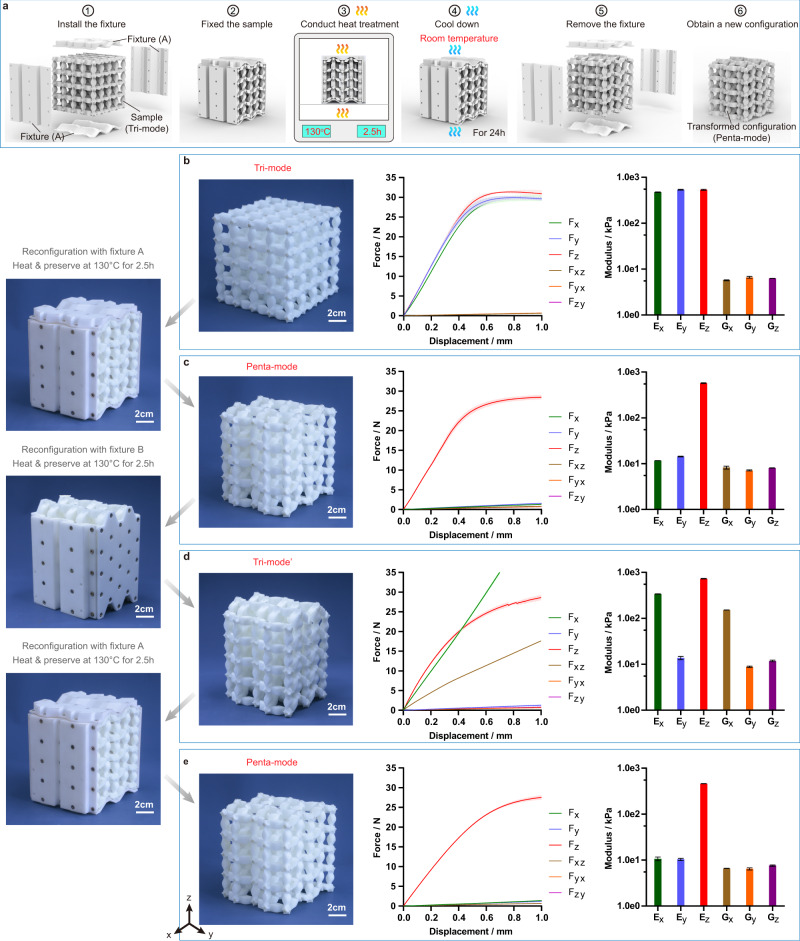
The reconfiguration and experimental validation of a 3D transformable metamaterial. **a** The schematic showing transformation steps with the fixture and heat treatment intermediate steps. **b** The 3D printed tri-mode metamaterial and its experimental displacement-force curves and calculated elastic moduli. Lines: mean; shading: one standard deviation. **c** The reconfigured penta-mode metamaterial and its experimental displacement-force curves and calculated elastic moduli. **d** The reconfigured alternative tri-mode (tri-mode’) metamaterial and its experimental displacement-force curves and calculated elastic moduli. **e** The penta-mode metamaterial reconfigured back from tri-mode’ configuration and its experimental displacement-force curves and calculated elastic moduli. Error bars represent standard deviation.

To explore the extremal property of the proposed 3D transformable metamaterial, a sample of the 4×4×4 tessellation is fabricated with Thermoplastic Polyurethanes (TPU, *E*_TPU_ = 48.9 MPa), by using Fused Deposition Molding (FDM) method. The TPU sample can transform into a target configuration by following a heat treatment process with a specific fixture, as shown in Fig. 2a. A transformation process of the selected three configurations of the sample, which are predicted to be two tri-mode and one penta-mode metamaterials via the numerical-based homogenization method, is demonstrated and the force-displacement response in each configuration is tested under different loadings (details can be found in Supplementary Note 4 and Supplementary Movie 3). Initially, the sample of an all-CE-configured tri-mode metamaterial (with three orthogonal shear deformations being zero modes) is fabricated by 3D printing along its body-diagonal direction to avoid the anisotropy. Its force-displacement responses are obtained under both compressive and shear loadings along the three orthogonal directions (Fig. 2b). With the help of the fixture A, tri-mode to penta-mode transformation of the sample is then performed following the transformation steps shown in Fig. 2a. Similar test is conducted on the configuration of penta-mode and the results are compared with those of the tri-mode, as shown in Fig. 2c. It can be found that the compressive moduli in all three orthogonal directions of the tri-mode are approximately two orders of magnitude larger than the shear moduli, verifying the existence of the three pure shear zero modes which are predicted with homogenization method. After transforming into the penta-mode, only the z-directional compressive modulus keeps (two orders of magnitude larger than any other compressive or shear moduli), which agrees well with the property of penta-mode. Furthermore, the sample is reconfigured into an alternative configuration of tri-mode (tri-mode’) by the fixture B to make the links along x-, y- and z-directions in the CF, PF and CE configurations, respectively. The experimental results demonstrate that the x and z directional compressive moduli as well as the shear moduli are two orders of magnitude larger than the rest three moduli, which confirms that it is a new form of tri-mode (Fig. 2d). Finally, with the fixture A again, we reconfigure the sample back into penta-mode, as shown in Fig. 2e, and its static experiments deliver the similar moduli as the one in Fig. 2c, which indicates that the reconfiguration is reversible. Noted that the slightly difference on the two penta-mode force-displacement curves is caused by the softening of the base material due to repeated heating (Supplementary Note 4). It also should be noted that although the transformation method is relatively simple, the requires for multiple fixtures and long heat treatment time can be an issue for real-time transformation among all seven types of extremal metamaterials.

**Fig. 3:**
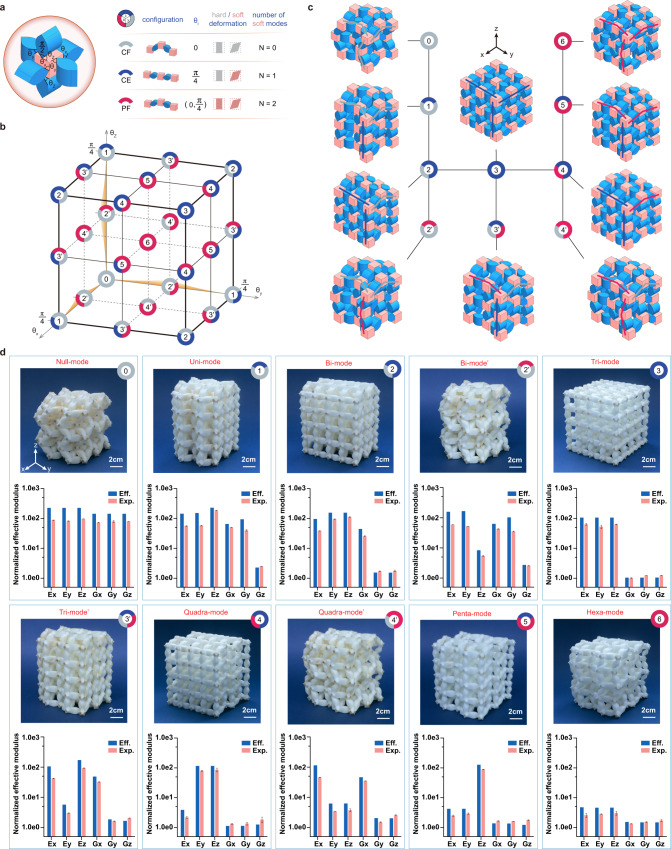
Schematic diagram of the 3D mechancial metamaterial’s transformability from null-mode to hexa-mode and its experimental validation. **a** The three basic configurations (gray-colored CF, blue-colored CE and red-colored PF), folding angles (*θ*_*i=x,y,z*_ can be 0, *π*/4 or in-between), the deformation types (hard or soft) and the corresponding number of zero modes. **b** A coordinate space which illustrates the 3D tessellation’s all possible combinations of the edge linkages and the corresponding types of the equivalent extremal metamaterials (0=null-mode, 1=uni-mode, 2=bi-mode, 3=tri-mode, 4=quadra-mode, 5=penta-mode, 6=hexa-mode). **c** The number of zero modes and the corresponding origami tessellations (CAD models) of the 3D transformable metamaterial. **d** Experimentally obtained and numerically calculated moduli of the ten configurations. Eff. represents effective properties obtained based on the homogenization method, and Exp. respresents experimental results obtained with the corresponding reconfigured tessellation, where error bars represent standard deviation.

Next, we will study this proposed metamaterial for its completed transformability covering null-mode (solid state) to hexa-mode (near-gaseous state). We first investigate a 2×2×2 tessellation, a representative unit cell of the 3D transformable metamaterial, whose possible configurations are similar with those of the aforementioned 2D case and therefore, can also be identified as CF, CE and PF with different folding angles *θ*_*i*_ (*i* = x, y, z) in three coordinates (Fig. 3a), where *θ*_*i*_ = 0, *θ*_*i*_ = *π*/4 and *θ*_*i*_ ∈ (0, *π*/4) correspond to the CF, CE and PF configurations, respectively. When the linkage along one orthogonal direction is in one of the three configurations, a number of zero modes *N*_*i*_ = 0, 1 or 2 can be identified, which contributes to the overall number of zero modes in the equivalent homogenized metamaterial, where all unit cells have the identical configuration. From a mechanism design perspective, the decoupled motions of the linkages, which form the three orthogonal directions, ensure that the superposition rule can be applied to calculate the overall number of zero modes in the metamaterial’s unit cell as:

1 $$N={N}_{x}+{N}_{y}+{N}_{z}$$

When the linkage in x, y, and z directions are all in CE configurations, we have *N*_*x*_ = *N*_*y*_ = *N*_*z*_ = 1, then the metamaterial can stand the axial load in three directions with three shear zero modes, thus it is a tri-mode with *N* = *N*_*x*_ + *N*_*y*_ + *N*_*z*_ = 3. When the linkages in x, y, z directions are changed into the CF, PF, and CE configurations, respectively, i.e., *N*_*x*_ = 0, *N*_*y*_ = 1, *N*_*z*_ = 2, then we have *N* = *N*_*x*_ + *N*_*y*_ + *N*_*z*_ = 0 + 1 + 2 = 3, which corresponds to the tri-mode’ (3′) metamaterial with three zero modes being axial deformation along y direction and shear deformations in y-z and x-y planes. With the motion ranges of *θ*_*i*_ (*i* = x, y, z) in all three coordinates being [0, *π*/4], a cubic working space forms in Fig. 3b. In the cube, the eight corner points are related to a specific type of zero mode metamaterial, e.g., when *θ*_*i*_ = 0, the metamaterial is a null-mode. Since the PF configuration corresponds to *θ*_*i*_ ∈ (0, *π*/4), all points along an edge of the cube (except the corner point) relate to a specific mode, e.g., when *θ*_*x*_ ∈ (0, *π*/4) and *θ*_*y*_ = *θ*_*z*_ = 0, the formed metamaterials are all bi-mode metamaterials. Similarly, all points on a surface of the cube (except the four edges) relate to a specific mode, e.g., when the points are on the *θ*_*x*_-*O*-*θ*_*y*_ surface, the formed metamaterials are all quadra-mode’ metamaterials. When the points inside the cube are selected, the formed metamaterials are all in hexa-mode. Hence, this cubic working space illustrates the relations between all possible configurations of the unit-cell linkages and their corresponding types of the equivalent extremal metamaterials. Since *N*_*i*_ ∈ [0, 1, 2] is for each linkage, the total number of zero modes is *N ∈* [0, 1, 2, …, 6], which means that all types of 3D extremal metamaterials, from null-mode to hexa-mode, can be physically realized by the proposed tessellation design.

Differing the three orthogonal directions in the tessellation is unnecessary and therefore, we can obtain ten distinctive extremal metamaterials, whose zero-mode numbers and the corresponding origami tessellations are demonstrated in Fig. 3c. Furthermore, the transformation among all ten tessellations can be realized by maneuvering the predefined folding angles (*θ*_*i*_) in each elementary linkage simultaneously (see Supplementary Movie 4). To analyze the ten metamaterials, we first choose the null-mode (*N* = 0, all-grey circle), tri-mode (*N* = 3, all-blue circle) and hexa-mode (*N* = 6, all-red circle), which also form the body diagonal of the cubic space in Fig. 3b. Close examination on the three tessellations reveals that all elementary linkages are in CF, CE or PF configuration and therefore, supports 3×0, 3×1 or 3×2 zero modes, respectively. It is noticed that the hexa-mode metamaterial cannot stand any type of loads and therefore, behaves like a nearly gaseous state. Next, we choose the ones marked by bi-colored circles, which are one uni-mode (1), two bi-modes (2, 2′), two quadra-modes (4, 4′) and one penta-mode (5). It can be found that all six metamaterials are constructed by combining linkages with two of the three configurations and therefore, support mixed compressive or/and pure shear zero modes. The only one with tri-colored circle is the tri-mode (3′) which is discussed in Fig. 2c. The detailed analysis of the corresponding zero modes via homogenization method can be found in Supplementary Note 5. In Fig. 3d, we perform experimental validations on the ten extremal metamaterials with a 3D printed 4x4x4 tessellation which can be transformed among all ten extremal metamaterials. A video demonstration of the tessellation transformation can be found in Supplementary Movie 5. Their effective moduli are obtained from the experimental measurements (see Supplementary Note 6) and the homogenization method (see Supplementary Note 5). Good agreement between the two results has been observed to prove the number of zero modes. We also conduct FE-based studies to validate the decoupled orthogonal mechanism design and evaluate the effects of different geometrical properties on the transformable metamaterial (see Supplementary Note 7). It can be concluded that the number of zero modes depends majorly on the folding angles, while both the thickness of the hinge *h*_0_ and the length of the rhombus *L*_0_ have little effect on the number of zero modes.

### Dynamic experiments and wave control analysis

**Fig. 4:**
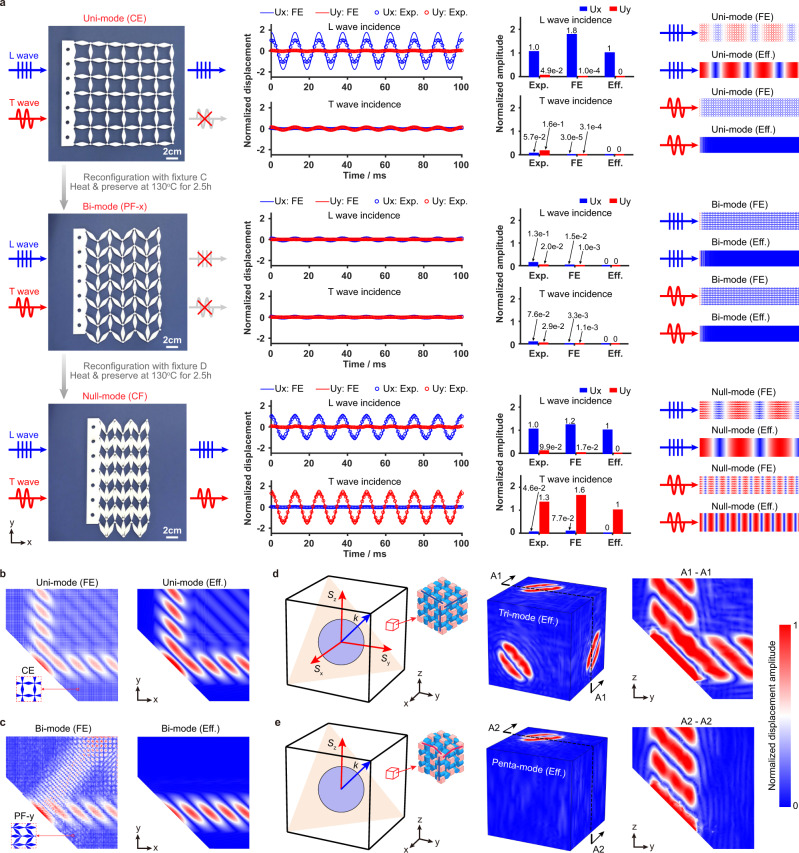
Dynamic experiments and wave-function programmability of the transformable metamaterial. **a** Dynamic experiment of 1D polarized elastic wave manipulation with the fabricated transformable metamaterial sample, validated by the numerical wave simulations on FE sample and equivalent effective metamaterial sample. **b, c** 2D wave-splitting and wave-filtering functions demonstrated with numerical wave simulations on the 2D FE metamaterial sample as well as on the equivalent effective metamaterial sample. **d, e** 3D wave-splitting and wave-filtering functions demonstrated with 3D homogenized effective metamaterial sample.

The customizable elasticity tensor of the transformable metamaterial can also lead to unconventional dynamic properties^10^. First, we experimentally illustrate the one-dimensional (1D) polarized elastic wave manipulation with the fabricated transformable metamaterial sample, as shown in Fig. 4a. The details of the dynamic experiment setup can be found in Supplementary Note 8. When the sample is in all-CE configuration and forms a uni-mode metamaterial, only longitudinal (L) wave can propagate. The measured x-directional displacement (Ux) for the L wave incidence is two orders of magnitude larger than the measured y-directional displacement (Uy) for the transverse (T) wave incidence, which coincides with the results of the numerical simulations on finite element (FE) metamaterial sample as well as the predictions with the homogenized effective metamaterial sample, as shown in the bar chart in Fig. 4a (details see Supplementary Note 8). For clearer illustrations on the two polarized (L and T) wave propagations, numerical wave simulations are also conducted on the length-extended FE sample as well as the equivalent effective metamaterial sample, where wave patterns can only be found in results of L wave incidence, which supports the results from the dynamic experiments. When the sample is reconfigured into PF configuration along x direction (PF-x), it forms a bi-mode metamaterial, which cannot support either L or T wave propagation. The experiment measurements as well as the FE and effective metamaterial simulations also agree well. When the sample is then reconfigured into CF configuration, it forms a null-mode metamaterial where both L and T wave can propagate, which is confirmed by the experiment, FE and effective metamaterial results.

More interestingly, when 2D and 3D wave propagations are considered, wave-function programmability can be achieved not only on the wave polarization but also on the wave direction. Take the 2D case for example. When a L wave beam is excited along the 45° direction (Fig. 4b), two 45° slant-polarized elastic waves with the same velocity are generated in the all-CE-configured tessellation, performing a unique 90° wave-splitting function. Such interesting wave control can be understood by analyzing the wave properties of the effective uni-mode metamaterial. Detailed analysis on the wave control equivalence between the origami tessellation and the homogenized metamaterial can be found in Supplementary Note 9. When the all-CE-configured tessellation is partially folded along the y direction (PF-y), only x-directional wave propagation is then supported (Fig. 4c), which agrees well with the wave behavior of the effective bi-mode metamaterial. The corresponding time-domain numerical simulations can be found in Supplementary Movie 6.

Since the wave control equivalence between the tessellations and effective metamaterials has been well validated for the 1D and 2D wave propagation cases, 3D wave-splitting as well as wave-filtering functions are demonstrated in an effective 3D metamaterial. Like the 2D case, with a L wave beam stimulated along the diagonal body direction of the cubic space, a three-wave-splitting phenomenon can be observed with the three slant-polarized waves propagating orthogonally along x, y, and z directions of the tri-mode metamaterial, respectively (Fig. 4d). By partially folding the 3D metamaterial along both x and y directions, a penta-mode configuration is reconfigured, which permits only z-directional wave propagation (Fig. 4e). By leveraging the dimension as well as the scale of the metamaterial system, one can realize more complicated programmable functionalities where different combinations of wave-direction and wave-polarization controls can be utilized.

## Discussion

In summary, we have designed a 3D transformable mechanical metamaterial to engineer extremal elasticity tensors ranging from solid to nearly gaseous state. Static and dynamic experiments are conducted to validate the transformable elasticity as well as the reprogrammable polarized wave control ability. Moreover, numerical demonstrations on the programmable wave functionality are also provided. This work brings a blueprint for rational design of 3D metamaterials whose static and dynamic effective properties are determined by the collective behavior of the atom-like mechanisms. By exploring different types of micro-linkages, a design toolbox can be constructed for customized metamaterials with broadband elastic wave manipulation capabilities. The reported design methodology also provides a versatile tool to engineer flexible metamaterials in various scales (if fabrication allows) and domains (such as mechanics, acoustics and photonics). To achieve the active and instant transformation of the constitutive properties as well as functionalities, the magneto-, electro- or thermo-mechanical coupled material can be integrated inside the fundamental cell of the metamaterial^46–49^, which accelerates the transformation process without requiring complex fixtures. Further exploration should also aim at unlocking the 3D metamaterial’s rich wave control abilities. In particular, the existence of zero-energy mode in the 3D structure can create topologically distinct classes, analogous to 3D topological insulators for polarized elastic waves^37^. Another direction of research could be explored based on this work involves the development of 3D complex materials that feature not only reprogrammable linear properties but also reversible nonlinearities that lead to switchable functionalities^50–52^.

## Methods

The details of the homogenization method and homogenization analysis of 2D and 3D extremal metamaterials are summarized in Supplementary Notes 1, 2 and 5. Kinematic property of the 3D metamaterial unit and the experiments and reconfiguration on 3D metamaterial are described Supplementary Notes 3, 4 and 6. The effects of geometrical parameters on the transformable metamaterial are analyzed in Supplementary Note 7. Finally, the dynamic experiments and wave control analysis of the transformable metamaterials are provided in Supplementary Notes 8 and 9.

## Supplementary information

Supplementary information

Description of Additional Supplementary Files

Supplementary Movie 1

Supplementary Movie 2

Supplementary Movie 3

Supplementary Movie 4

Supplementary Movie 5

Supplementary Movie 6

## Data Availability

All the data supporting the conclusions of this study are included in the article and the Supplementary Information file. Source data are provided with this paper.

## Source data

Source Data

## Code availability

The computer code and algorithm that support the findings of this study are available from the corresponding author on request.
