# Supplementary material for: Engineering zero modes in transformable mechanical metamaterials
Source: Nat Commun. 2023 Mar 7;14:1266. doi: 10.1038/s41467-023-36975-2 (PMC9992356; doi:10.1038/s41467-023-36975-2)
Supplement: Supplementary file 1 — Supplementary information [file 41467_2023_36975_MOESM1_ESM.pdf]

Supplementary Information for

Engineering Zero Modes in Transformable Mechanical Metamaterials

Zhou Hu[1 †], Zhibo Wei[2 †], Kun Wang[1], Yan Chen[2,3], Rui Zhu[1,4*], Guoliang Huang[5], and Gengkai Hu[1]*

[1]*School of Aerospace Engineering, Beijing Institute of Technology, Beijing 100081, China*

[2]*School of Mechanical Engineering, Tianjin University, Tianjin 300350, China*

[3]*Key Laboratory of Mechanism Theory and Equipment Design of Ministry of Education, Tianjin University, Tianjin, 300350, China*

[4]*Beijing Institute of Technology Chongqing Innovation Center, Chongqing 401120, China*

[5]*Department of Mechanical and Aerospace Engineering, University of Missouri, Columbia, MO 65211, USA*

Supplementary Notes

Supplementary Note 1. Homogenization method and classification of the designed metamaterials.

A. Numerical-based homogenization method via the Cauchy-Born hypothesis

B. Classification of the extremal metamaterials

Supplementary Note 2. Homogenization analysis of 2D metamaterials.

Supplementary Note 3. Kinematic property of the 3D metamaterial unit.

Supplementary Note 4. Static experiments on 3D reconfigurable metamaterials.

Supplementary Note 5. Homogenization analysis of 3D metamaterials.

Supplementary Note 6. Effective compressive and shear moduli of the ten configurations.

Supplementary Note 7. Effects of different geometrical parameters.

Supplementary Note 8. Dynamic experiments on the transformable metamaterial.

Supplementary Note 9. 2D and 3D wave analysis of transformable metamaterials.

---

† These authors contributed equally to this work.

* yan_chen@tju.edu.cn；

* ruizhu@bit.edu.cn；

Supplementary Note 1. Homogenization and classification of the designed metamaterials

A. Numerical-based homogenization method via the Cauchy-Born hypothesis

The homogenization theory [1-3] establishes the relationship between the microstructures and the macroscopic properties of the metamaterial by assuming that there is an equivalent continuous medium which exhibits the same macroscopic behavior as the metamaterial. Due to the complexity of the microstructures in the proposed metamaterial, a numerical-based homogenization method is developed with the help of commercial finite element (FE) method software to calculate the effective elasticity tensors of the metamaterials.

First, the area of a unit cell in 2D metamaterial can be defined as $S = |(\boldsymbol{a}_1 \times \boldsymbol{a}_2)|$, where $\boldsymbol{a}_1$ and $\boldsymbol{a}_2$ are the two translation vectors of the unit cell. For the 3D metamaterial, the volume of a unit cell is defined as $V = |\boldsymbol{a}_3 \cdot (\boldsymbol{a}_1 \times \boldsymbol{a}_2)|$, where $\boldsymbol{a}_i$ $(i = 1,2,3)$ represents the translation vector. Periodic boundary conditions (PBCs) are imposed on the unit cell's boundary with the Cauchy-Born hypothesis being considered. Therefore, the microscopic displacement deformations at boundary can be expressed as a function of a macroscopic strain field $\mathbf{E}$ [2,4]

$$\mathbf{u}(\mathbf{X_b} + \mathbf{a}) = \mathbf{u}(\mathbf{X_b}) + \mathbf{E} \cdot \mathbf{a} \tag{1}$$

where $\mathbf{u}(\mathbf{X_b})$ is the displacement at a node point $\mathbf{X_b}$ located on the boundary of the meshed unit cell, and $\boldsymbol{a}$ represents the translation vector between two nearby unit cells. It is worth noting that these constraints automatically guarantee traction continuity on the unit cell's boundary. Next, the strain energy of the metamaterial unit cell U_0 and that of an effective medium with the same area/volume U_{eff} can be calculated directly with the help of a commercial FE software. Finally, following the assumption of homogenization stating that the strain energy obtained from a metamaterial's unit cell is equal to that obtained from an effective medium, namely

$$U_0 = U_{eff} = \frac{V}{2}\mathbf{E}\colon \mathbf{C}^{eff}\colon \mathbf{E} \tag{2}$$

the components of the effective elasticity tensor $\mathbf{C}^{eff}$ can be determined [5].

By recognizing the symmetry of the elasticity tensor, 6 (or 21) independent components for the 2D (or 3D) case are unknown, and we need to specify at least 6 (or 21) different types of macroscopic strain fields to determine the effective elasticity tensor of a 2D (or 3D) metamaterial. Specifically, we specify 6 types of macroscopic strain fields as two uniaxial, one biaxial, one shear, and two combinations of uniaxial and shear strains for the 2D case, while we choose 21 types of macroscopic strain fields as three uniaxial, three shears, three biaxial, nine combinations of uniaxial and shear, and three combinations of shear strains [5].

B. Classification of the extremal metamaterials

It is well known that general Hooke's law states the linear relationship between stress and strain, as $\boldsymbol{\sigma} = \mathbf{C}: \boldsymbol{\varepsilon}$, where $\mathbf{C}$ represents the fourth-order elasticity tensor which can be represented as a stiffness matrix **c,** a positive definite symmetric $n \times n$ matrix ($n = 3$ for 2D elasticity and $n = 6$ for 3D elasticity). When elastic symmetry is considered [6], general Hooke's law can then be rewritten as

$$\vec{\boldsymbol{\sigma}} = \mathbf{c}\vec{\boldsymbol{\varepsilon}} \tag{3}$$

where the stress $\vec{\boldsymbol{\sigma}}$ and strain $\vec{\boldsymbol{\varepsilon}}$ are n-element column matrixes. Mathematically, the stiffness matrix $\mathbf{c}$ has n real eigenvalues $\lambda^{(p)}$ with their corresponding mutually orthogonal eigenvectors, $\vec{\mathbf{S}}^{(p)}$. One can have

$$\lambda^{(p)}\vec{\mathbf{S}}^{(p)} = \mathbf{c}\vec{\mathbf{S}}^{(p)} \tag{4}$$

where $(\vec{\mathbf{S}}^{(p)})^T\vec{\mathbf{S}}^{(q)} = \delta_{pq},\ p, q = 1,2, \dots, n.$ δ_{pq} is the Kronecker delta. Comparing Eq. (3) with Eq. (4), the eigenvalues and eigenvectors of the stiffness matrix are related to the principal stiffnesses and strains, respectively.

Interestingly, if an eigenvalue $\lambda^{(p)}$ goes to zero, the corresponding principal stiffness of the material also turns into zero, and therefore, no stress is generated when deformation is applied along the principal direction. As a result, a zero mode can be identified for the material. By counting the number of zero eigenvalues, the number of existing zero modes can be found accordingly and we can categorize all elastic

materials into four types of extremal metamaterials from null-mode to tri-mode for 2D cases or seven types of extremal metamaterials from null-mode to hexa-mode for 3D cases [7].

Supplementary Note 2. Homogenization analysis of 2D metamaterials.

Here, a representative unit cell for 2D tessellations is shown in Supplementary Fig. 1. For each unit cell, the side length and the minimal angle of the rhombus are $L_0 = 5$ mm and $\theta_0 = \pi/12$, respectively, and the radius at the hinge is $r_0 = 0.5$ mm. Two angles denoted by θ_x and θ_y are defined to distinguish different configurations. Here, $\theta_x = 0, \theta_y = \pi/3$ corresponds to the CF configuration, $\theta_x = \theta_y = 0$ corresponds to the CE configuration, $\theta_x = 0, \theta_y = \pi/4$ (or $\theta_x = \pi/4, \theta_y = 0$) corresponds to the PF-y (or PF-x) configuration, and $\theta_x = \theta_y = \pi/8$ corresponds to the PF configuration in both directions.

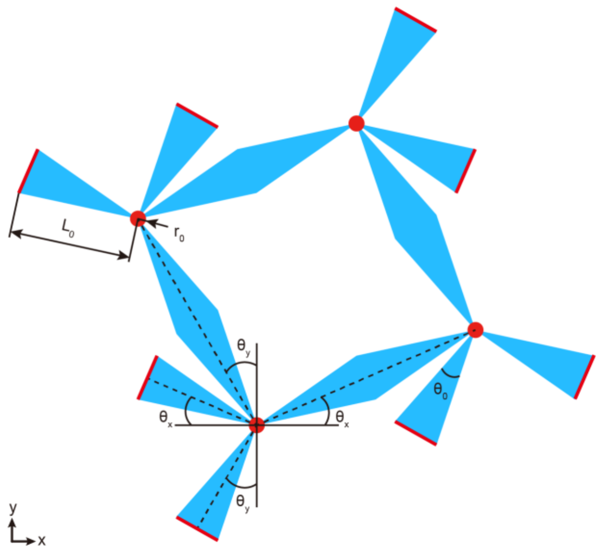

Supplementary Fig. 1 The schematic unit cell for 2D tessellations.

With the aforementioned numerical-based homogenization method, we can calculate the effective elasticity tensors of all four types of 2D metamaterials shown in Fig. 1a in the main text, and they are rewritten as the form of the stiffness matrix $\mathbf{c}^{eff}$. Due to the more practical weakened connections are applied to replace the ideal revolute joints, the corresponding eigenvalues of the effective stiffness matrix may not accurately be zero. With the eigenvalues less than 1.0e-3 being recognized as the ones corresponding

to the zero modes, the classification for the four 2D metamaterial unit cells are shown in Supplementary Table 1.

For the first configuration, three eigenvalues of the effective stiffness matrix are larger than 1.0e-3 and therefore, no zero mode can be found, which indicates that it is a null-mode metamaterial. For the second configuration, only one eigenvalue (7.0e-5) is smaller than 1.0e-3 and therefore, a uni-mode metamaterial is identified with the only zero mode being shear deformation. For the third configuration, only one eigenvalue (1.4e-1) is larger than 1.0e-3 and therefore, a bi-mode metamaterial is identified with the shear deformation and the uniaxial deformation along the y-direction being related to the two zero modes, respectively. Moreover, this type of bi-mode metamaterial is different from the previously reported bi-mode metamaterial which only supports hydrostatic pressure. For the last configuration, all three eigenvalues are smaller larger than 1.0e-3 and therefore, a tri-mode metamaterial is identified.

Unit cell	$\mathbf{c}^{eff} / E_0$	Eigenvalues	Zero mode	Classification
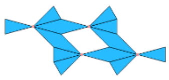	$\begin{bmatrix} 2.8e\text{-}1 & 3.0e\text{-}2 & 0 \\ & 2.5e\text{-}1 & 0 \\ sym & & 1.9e\text{-}2 \end{bmatrix}$	1.9e-2<br>2.2e-1<br>3.0e-1	Null	Null-mode
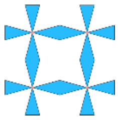	$\begin{bmatrix} 1.0e\text{-}1 & 6.7e\text{-}3 & 0 \\ & 1.0e\text{-}1 & 0 \\ sym & & 7.0e\text{-}5 \end{bmatrix}$	**7.0e-5**<br>9.5e-2<br>1.1e-1	$\begin{pmatrix} 0 \\ 0 \\ 1 \end{pmatrix}$	Uni-mode
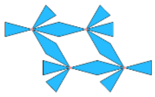	$\begin{bmatrix} 1.4e\text{-}1 & -2.0e\text{-}4 & 0 \\ & 1.9e\text{-}4 & 0 \\ sym & & 1.0e\text{-}4 \end{bmatrix}$	**1.0e-4**<br>**1.9e-4**<br>1.4e-1	$\begin{pmatrix} 0 \\ 1 \\ 0 \end{pmatrix}, \begin{pmatrix} 0 \\ 0 \\ 1 \end{pmatrix}$	Bi-mode
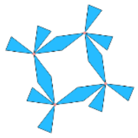	$\begin{bmatrix} 9.1e\text{-}4 & 0 & 0 \\ & 9.1e\text{-}4 & 0 \\ sym & & 8.0e\text{-}5 \end{bmatrix}$	**8.0e-5**<br>**9.1e-4**<br>**9.1e-4**	$\begin{pmatrix} 1 \\ 0 \\ 0 \end{pmatrix}, \begin{pmatrix} 0 \\ 1 \\ 0 \end{pmatrix}, \begin{pmatrix} 0 \\ 0 \\ 1 \end{pmatrix}$	Tri-mode

Supplementary Table 1 The normalized effective stiffness matrix, the calculated eigenvalues, the corresponding zero mode and the classification of each 2D metamaterial. All eigenvalues are normalized by the young's modulus of the constituent E_0.

Supplementary Note 3. Kinematic property of the 3D metamaterial unit.

As shown in Supplementary Fig. 2a, the 3D unit cell consists of eight cubes and twelve rhomboids. From a mechanism design perspective, one can take the cubes and rhomboids as links, and take the rotation connections between the cube and rhomboid as revolute joints. Hence, the mechanical model of such unit cell in Supplementary Fig. 2b consists of 8 corner links, 12 edge links and 24 revolute joints. In details, joints $B1_a$, $B1_b$, $B2_a$, $B2_b$ marked by orange color along the z-axis form the 4-bar linkage in x-y plane on the top side of the unit. The other 4-bar linkage in x-y plane is formed with joints $D1_a$, $D1_b$, $D2_a$, $D2_b$ on the bottom side of the unit. Similarly, joints $A1_a$, $A1_b$, $C1_a$, $C1_b$ and joints $A2_a$, $A2_b$, $C2_a$, $C2_b$, marked by blue color, form two 4-bar linkages in x-z plane, while joints $E1_a$, $E1_b$, $E2_a$, $E2_b$ and joints $F1_a$, $F1_b$, $F2_a$, $F2_b$, marked by red color, form two 4-bar linkages in y-z plane.

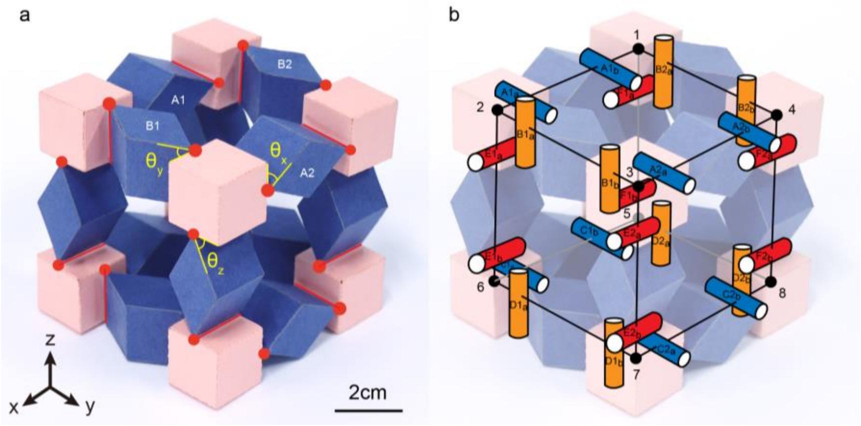

Supplementary Fig. 2 The unit cell and its mechanism schematic. a. The prototype of the 3D unit cell with 8 cubes and 12 rhomboids. **b.** The mechanical model of the 3D unit cell with 8 corner links, 12 edge links and 24 revolute joints.

The planar 4-bar linkage is one of the simplest linkages with four links and four revolute joints to form a closed loop. Once one of four links is fixed to the ground, the linkage becomes one-degree-of-freedom (1-DoF) system, i.e., the rotation of any one revolute joint will decide the rotations of the rest three joints kinematically. As a result, the configuration of the linkage is known. For our unit cell, the two linkages in the same directional set always have the simultaneous motion as their four respective joints are collinear, i.e., $A1_a$ and $A2_a$ are collinear, etc., so these two linkages form a 1-DoF system. There are three such systems along three coordinate axes, respectively. Their

motions are independent in each direction and therefore, a unit with decoupled motions of six 4-bar linkages in three orthogonal directions can be obtained subsequently. Hence, the unit has three degrees of freedom.

Since the unit cell is a 3-DoF system, the workspace of the unit cell is distributed in the entire three-dimensional space, and the boundary of the workspace is defined by the maximum rotation angle of the rhomboids in a single direction, which can be tuned by changing the angle of the rhomboids accordingly. Taking the linkage $A2_a$ -$A2_b$-$C2_a$-$C2_b$ as an example, we can find that when the small angle of the rhombus is $\pi/2$, θ_x can rotate in the range of 0 to $\pi/2$. At 0 or $\pi/2$, the 4-bar linkage reaches the limiting position while at $\pi/4$, the rhombus stays in the middle position, which makes the unit in a cube profile. Hence, we only consider the half motion range with $\theta_x \in (0, \pi/4)$, as the two halves are symmetry about the middle position. The same rule applied to θ_y and θ_z and therefore, the working space of this 3-DoF unit cell can be described as a cubic space with a side length of $\pi/4$, as shown in Supplementary Fig. 3. By controlling the rotation angle of the rhomboids in different directions, the structure can be moved to any point in the workspace, and the movement path is also diverse. Three motion paths starting from the origin are chosen to show the structural transformation capabilities, including curved paths along the coordinate axis, along the diagonal of the cube surface, and across the workspace. Under the first path, by setting $\theta_y = \theta_z = 0$, while changing θ_x from 0 to $\pi/8$ and then to $\pi/4$, the unit traverses any point on the x coordinate axis. In the second path, by setting $\theta_y = 0$, while changing θ_x and θ_z in equal proportions, the unit moves diagonally from the origin to another vertex on the x-z plane. Under the third path, the unit cell passes through the coordinate origin (0, 0, 0), coordinate point ($\pi/12$, $\pi/8$, $\pi/8$), and coordinate point ($\pi/4$, $\pi/4$, $\pi/8$) successively.

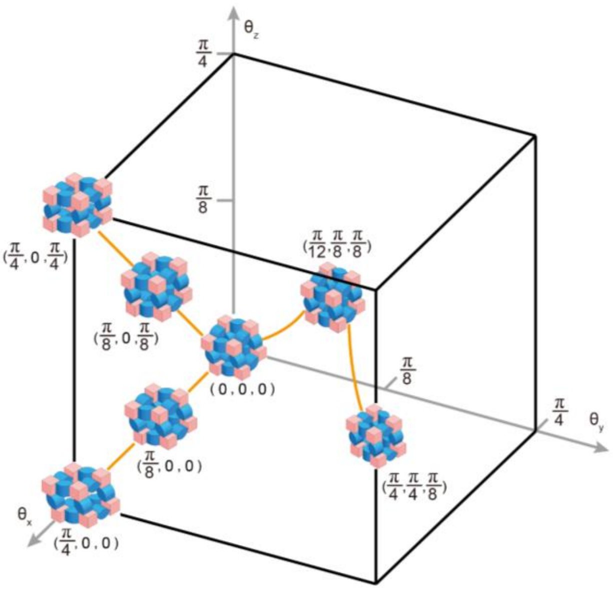

Supplementary Fig. 3 Workspace representation and three possible motion paths.

Supplementary Note 4. Static experiments on 3D reconfigurable metamaterials.

The experimental samples of the metamaterial are manufactured using a 3D printer (Raise 3D Pro2). For the printing process of metamaterial with complex geometry, we choose thermoplastic polyurethanes (TPU) as the modelling material and water-soluble PVA as the supporting material (holds all overhanging parts and can be removed later). In order to reduce the anisotropy of the printed sample, the effect of the printing direction is considered and the 4×4×4 sample (in its initial all-CF configuration) is printed along its body-diagonal direction, as shown in Supplementary Fig. 4.

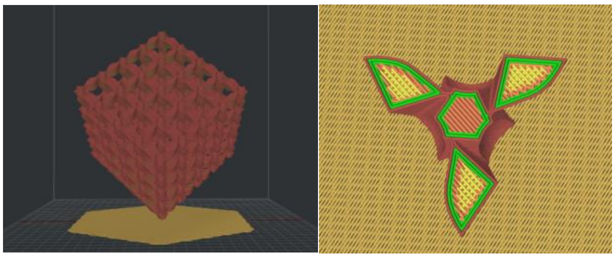

Supplementary Fig. 4 The 3D printing setup for the 4×4×4 sample in all-CF configuration to be printed along its body-diagonal direction.

With the help of the numerical-based homogenization method, three configurations of the sample are investigated and are predicted to be tri-mode, tri-mode’ (an alternative tri-mode configuration) and penta-mode, as shown in Supplementary Fig. 5.

Unit cell	$\mathbf{c}^{eff} / E_0$	Eigenvalues	Zero mode	Classification
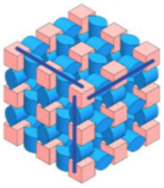	$\begin{bmatrix} 4.0e\text{-}2 & 1.0e\text{-}3 & 1.0e\text{-}3 & 0 & 0 & 0 \\ & 4.0e\text{-}2 & 1.0e\text{-}3 & 0 & 0 & 0 \\ & & 4.0e\text{-}2 & 0 & 0 & 0 \\ & & & 3.6e\text{-}5 & 0 & 0 \\ & sym & & & 3.6e\text{-}5 & 0 \\ & & & & & 3.6e\text{-}5 \end{bmatrix}$	3.6e-5<br>3.6e-5<br>3.6e-5<br>3.9e-2<br>3.9e-2<br>4.2e-2	$\begin{pmatrix}0\\0\\0\\1\\0\\0\end{pmatrix}, \begin{pmatrix}0\\0\\0\\0\\1\\0\end{pmatrix}, \begin{pmatrix}0\\0\\0\\0\\0\\1\end{pmatrix}$	Tri-mode<br>(3)
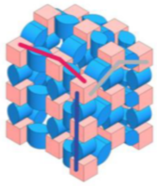	$\begin{bmatrix} 2.0e\text{-}2 & 0 & 7.6e\text{-}4 & 0 & 0 & 0 \\ & 2.9e\text{-}4 & 0 & 0 & 0 & 0 \\ & & 5.1e\text{-}2 & 0 & 0 & 0 \\ & & & 4.5e\text{-}5 & 0 & 0 \\ & sym & & & 4.1e\text{-}3 & 0 \\ & & & & & 4.9e\text{-}5 \end{bmatrix}$	4.5e-5<br>4.9e-5<br>2.9e-4<br>4.1e-3<br>2.0e-2<br>5.1e-2	$\begin{pmatrix}0\\0\\0\\1\\0\\0\end{pmatrix}, \begin{pmatrix}0\\0\\0\\0\\0\\1\end{pmatrix}, \begin{pmatrix}0\\1\\0\\0\\0\\0\end{pmatrix}$	Tri-mode'<br>(3')
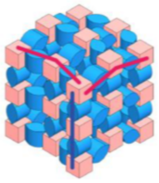	$\begin{bmatrix} 2.5e\text{-}4 & 0 & 0 & 0 & 0 & 0 \\ & 2.5e\text{-}4 & 0 & 0 & 0 & 0 \\ & & 4.3e\text{-}2 & 0 & 0 & 0 \\ & & & 3.9e\text{-}5 & 0 & 0 \\ & sym & & & 4.3e\text{-}5 & 0 \\ & & & & & 4.3e\text{-}5 \end{bmatrix}$	3.9e-5<br>4.3e-5<br>4.3e-5<br>2.5e-4<br>2.5e-4<br>4.3e-2	$\begin{pmatrix}0\\0\\0\\1\\0\\0\end{pmatrix}, \begin{pmatrix}0\\0\\0\\0\\0\\1\end{pmatrix}, \begin{pmatrix}0\\0\\0\\0\\1\\0\end{pmatrix}, \begin{pmatrix}1\\0\\0\\0\\0\\0\end{pmatrix}, \begin{pmatrix}0\\1\\0\\0\\0\\0\end{pmatrix}$	Penta-mode<br>(5)

Supplementary Fig. 5 The normalized effective stiffness matrix, the calculated eigenvalues, the corresponding zero mode for selected three configurations of the sample. All eigenvalues are normalized by the young's modulus of the constituent E_0.

A three-step reconfiguration path is chosen from tri-mode to penta-mode, then to tri-mode' (3' in Fig. 3), and return to penta-mode. Nylon fixtures A and B are fabricated by 3D printing (Selective Laser Sintering) and are used for reconfiguring the sample into penta-mode and tri-mode' configurations as displayed in Supplementary Fig. 6a and 6b, respectively.

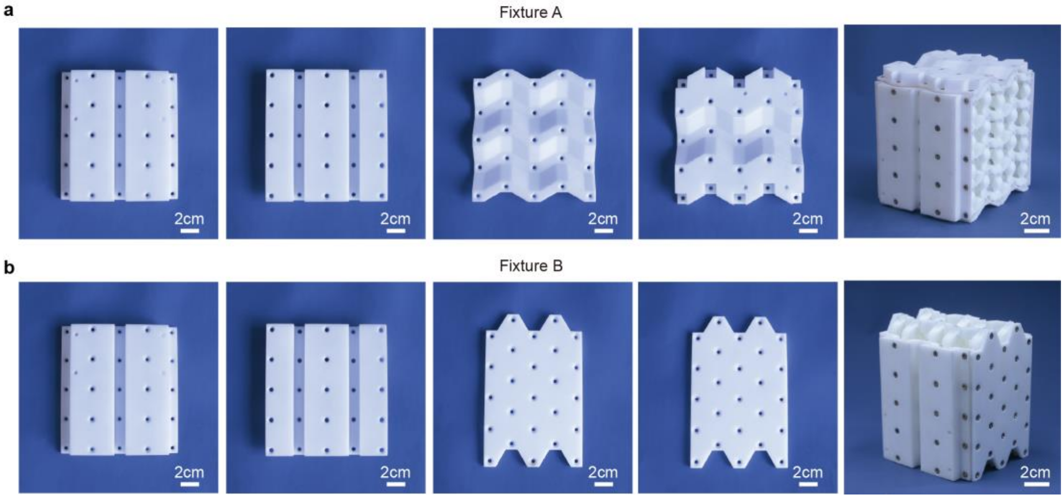

Supplementary Fig. 6 Photograph of fixtures used to reconfigure the sample. a. Fixture A for the penta-mode configuration. **b.** Fixture B for the tri-mode' configuration.

To study the mechanical properties of the sample, the compression and shear tests are conducted on INSTRON 5982 High Force Universal Testing Machine. A photograph of the experiment setup is shown in Supplementary Fig. 7.

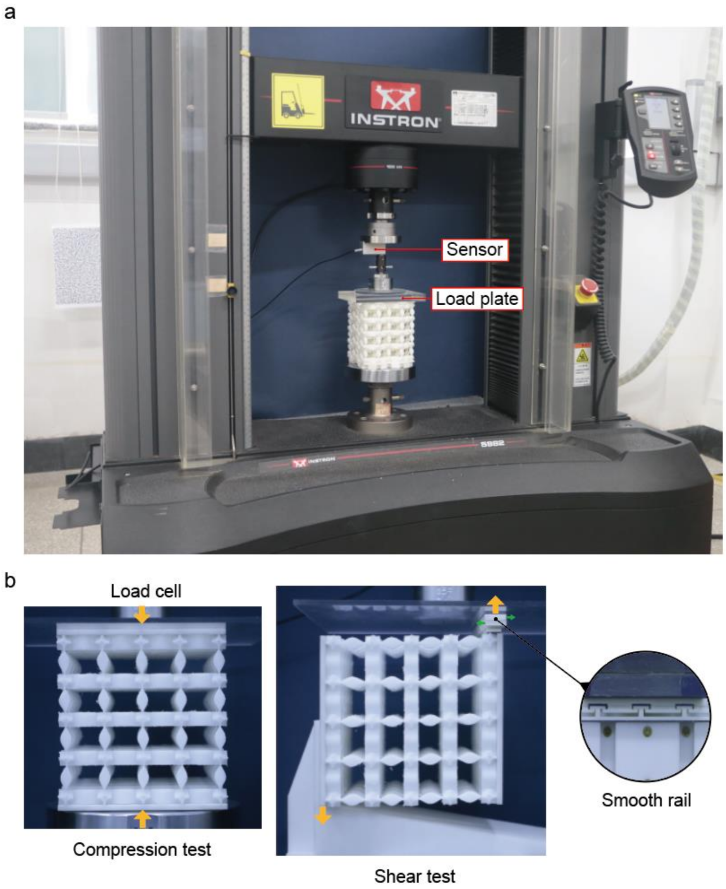

Supplementary Fig. 7 Experiment setup for the compression and shear tests. a. Photograph of the INSTRON testing platform. **b.** The setup of base and rail used for compression and shear tests.

First, compression tests are conducted to obtain the force-displacement curves along the three orthogonal directions. Considering the small deformation, the Young's moduli along the three directions can be calculated from the force-displacement curves as,

$$E_\alpha = \frac{\sigma_\alpha}{\varepsilon_\alpha} = \frac{F_\alpha / A_\alpha}{u_\alpha / L_\alpha} \tag{5}$$

where $\alpha = x, y, z$, σ_α and ε_α represent the compressive stress and axial strain, u_α and F_α represent the applied displacement and measured force, respectively. L_α and A_α are the width and the cross-section area of the sample, respectively. Second, shear tests are conducted and the sample is rotated 90 degrees with one end being fixed on the base while the other end being connected to the loading unit, as shown in

Supplementary Fig. 7. To ensure pure shear deformation, a section of rail is designed and installed between the loading unit and the end of the sample to minimize unwanted bending moment. Hence, the relationship between the shear force and the corresponding displacement can be obtained and therefore, the shear moduli can be derived from [8]

$$G_\alpha = \frac{\tau_{\alpha\beta}}{\gamma_{\alpha\beta}} = \frac{F_{\alpha\beta}/A_\alpha}{u_\beta/L_\alpha} \tag{6}$$

Where $\tau_{\alpha\beta}$ and $\gamma_{\alpha\beta}$ represent the shear stress and strain, $F_{\alpha\beta}$ represents the measured shear force, and u_β represents the corresponding displacement. The initial parts of force-displacement curves with the displacements from 0 to 0.2mm are selected to calculate the Young's moduli and shear moduli. Moduli obtained from the experimental results are shown in Supplementary Table 2.

	E_x/kPa	E_y/kPa	E_z/kPa	G_x/kPa	G_y/kPa	G_z/kPa
Tri-mode	4.7e2	5.4e2	5.4e2	5.7	6.6	6.3
Penta-mode	1.7e1	1.4e1	5.7e2	8.3	7.1	8.0
Tri-mode'	3.4e2	1.4e1	7.3e2	1.5e2	8.8	1.7e1
Penta-mode	1.1e1	1.0e1	4.6e2	6.6	6.5	7.6

Supplementary Table 2 Moduli of sample in different configurations.

Additionally, both x-directional compression and tension tests are conducted for the sample in the tri-mode' configuration, as shown in Supplementary Fig. 8a. In this case, the sample is in the CF configuration along the x direction, where the cubes and rhomboids are bonded with adhesives which can be dissolved with degummant when necessary. As shown in the figure, under the two loading methods, the obtained force-displacement curves are relatively consistent and linear, and only small difference can be found.

To further evaluate the reversibility of the TPU material under thermal treatment, tensile tests have been conducted on a dog-bone specimen made with TPU, which was repeatedly thermally treated for four rounds. After each round of treatment, which is the same thermal treatment as that given to the 3D tessellation sample, a tensile test was

performed and the results of the initially printed specimen and the specimen after four rounds of thermal treatments are shown in Supplementary Fig. 8b. It can be found that the obtained material modulus was softened in the first two rounds, but stabilized afterwards, which indicates that the TPU material is suitable for reversible thermal treatment.

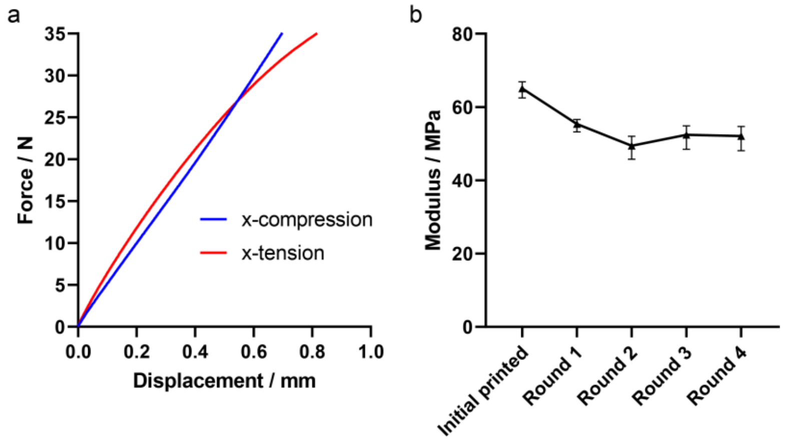

Supplementary Fig. 8 a. The x-direction compression and tension test results for the sample in the tri-mode' configuration. **b.** Tensile test results of a dog-bone specimen at initial printed state and after four thermal treatment rounds for 2.5 hours at 130°C. Error bars represent standard deviation.

Supplementary Note 5. Homogenization analysis of 3D metamaterials.

We study the ten types of 3D metamaterials shown in Fig. 3c in the main text via the homogenization method. Noted that weaken connections are also considered instead of ideal revolute joints, so the corresponding eigenvalues of the effective stiffness matrix are not accurately zero. With the eigenvalues less than 1.0e-3 being recognized as the ones corresponding to the zero modes, the normalized effective stiffness matrix, the calculated eigenvalues, the corresponding zero mode and the classification of each 3D metamaterial are shown in Supplementary Fig. 9.

For the first configuration, $\theta_i = 0$ ($i = x, y, z$), all eigenvalues of the effective stiffness matrix are larger than 1.0e-3 and therefore, no zero mode can be found, which indicates that it is a null-mode metamaterial. For the second configuration, $\theta_z = \pi/4$ while $\theta_x = \theta_y = 0$, only one eigenvalue is smaller than 1.0e-3 and therefore, a uni-mode metamaterial is identified with the only zero mode being shear deformation in y-z plane. For the third

configuration, $\theta_z = \theta_y = \pi/4$ while $\theta_x = 0$, two eigenvalues are smaller than 1.0e-3 and therefore, a bi-mode metamaterial is identified with the two zero modes being the two shear deformations in y-z and x-y planes. For the fourth configuration, $\theta_x = \theta_y = 0$ while $\theta_z = \pi/8$, two eigenvalues are smaller than 1.0e-3 and therefore, an alternative bi-mode (bi-mode') metamaterial is identified with the two zero modes being the axial deformation along the z direction and the shear deformation in y-z plane. For the fifth configuration, $\theta_i = \pi/4$ ($i = x, y, z$), three eigenvalues are smaller than 1.0e-3 and therefore, a tri-mode metamaterial is identified with the three zero modes being the shear deformations in x-y, y-z and x-z planes. For the sixth configuration, $\theta_x = 0$, $\theta_y = \pi/8$ and $\theta_z = \pi/4$, three eigenvalues are smaller than 1.0e-3 and therefore, an alternative tri-mode (tri-mode') metamaterial is identified with three zero modes being the axial deformation along the z direction and the two shear deformations in y-z and x-y planes. For the seventh configuration, $\theta_x = \pi/8$ while $\theta_y = \theta_z = \pi/4$, four eigenvalues are smaller than 1.0e-3 and therefore, a quadra-mode metamaterial is identified with four zero modes being the three shear deformations and the axial deformation along the x direction. For the eighth configuration, $\theta_x = 0$ while $\theta_y = \theta_z = \pi/8$, four eigenvalues are smaller than 1.0e-3 and therefore, an alternative quadra-mode (quadra-mode') metamaterial is identified with four zero modes being the two shear deformations in x-y and y-z planes and the two axial deformations along the y and z directions. For the ninth configuration, $\theta_x = \theta_y = \pi/8$ while $\theta_z = \pi/4$, five eigenvalues are smaller than 1.0e-3 and therefore, a penta-mode metamaterial is identified with five zero modes being the three shear deformations and the two axial deformations along the x and y directions. For the last configuration, $\theta_i = \pi/8$ ($i = x, y, z$), all eigenvalues are smaller than 1.0e-3 and therefore, a hexa-mode metamaterial is identified with all axial and shear deformations being zero modes. It is the 3D version of the 2D tri-mode metamaterial.

Unit cell	$\mathbf{c}^{eff} / E_0$	Eigenvalues	Zero mode	Classification
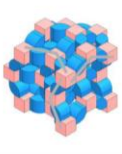	$\begin{bmatrix} 2.9e\text{-}2 & 4.0e\text{-}4 & 4.0e\text{-}4 & 0 & 0 & 0 \\ & 2.9e\text{-}2 & 4.0e\text{-}4 & 0 & 0 & 0 \\ & & 2.9e\text{-}2 & 0 & 0 & 0 \\ & & & 8.8e\text{-}3 & 0 & 0 \\ & sym & & & 8.8e\text{-}3 & 0 \\ & & & & & 8.8e\text{-}3 \end{bmatrix}$	8.8e-3<br>8.8e-3<br>8.8e-3<br>2.8e-2<br>2.8e-2<br>2.9e-2	Null	Null-mode<br>(0)
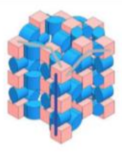	$\begin{bmatrix} 2.3e\text{-}2 & 3.2e\text{-}4 & 8.4e\text{-}4 & 0 & 0 & 0 \\ & 2.3e\text{-}2 & 9.3e\text{-}4 & 0 & 0 & 0 \\ & & 5.8e\text{-}2 & 0 & 0 & 0 \\ & & & 5.2e\text{-}5 & 0 & 0 \\ & sym & & & 4.7e\text{-}3 & 0 \\ & & & & & 7.1e\text{-}3 \end{bmatrix}$	**5.2e-5**<br>4.7e-3<br>7.1e-3<br>2.3e-2<br>2.4e-2<br>5.8e-2	$\begin{pmatrix}0\\0\\0\\1\\0\\0\end{pmatrix}$	Uni-mode<br>(1)
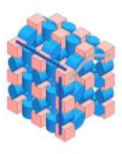	$\begin{bmatrix} 1.9e\text{-}2 & 8.9e\text{-}4 & 7.5e\text{-}4 & 0 & 0 & 0 \\ & 4.8e\text{-}2 & 1.3e\text{-}3 & 0 & 0 & 0 \\ & & 4.8e\text{-}2 & 0 & 0 & 0 \\ & & & 4.2e\text{-}5 & 0 & 0 \\ & sym & & & 3.9e\text{-}3 & 0 \\ & & & & & 4.2e\text{-}5 \end{bmatrix}$	**4.2e-5**<br>**4.2e-5**<br>3.9e-3<br>1.9e-2<br>4.7e-2<br>4.9e-2	$\begin{pmatrix}0\\0\\0\\1\\0\\0\end{pmatrix}, \begin{pmatrix}0\\0\\0\\0\\0\\1\end{pmatrix}$	Bi-mode<br>(2)
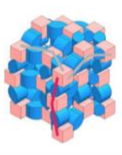	$\begin{bmatrix} 2.4e\text{-}2 & 3.2e\text{-}4 & 0 & 0 & 0 & 0 \\ & 2.4e\text{-}2 & 0 & 0 & 0 & 0 \\ & & 3.5e\text{-}4 & 0 & 0 & 0 \\ & & & 5.9e\text{-}5 & 0 & 0 \\ & sym & & & 4.7e\text{-}3 & 0 \\ & & & & & 7.5e\text{-}3 \end{bmatrix}$	**5.9e-5**<br>**3.5e-4**<br>4.7e-3<br>7.5e-3<br>2.4e-2<br>2.5e-2	$\begin{pmatrix}0\\0\\0\\1\\0\\0\end{pmatrix}, \begin{pmatrix}0\\0\\1\\0\\0\\0\end{pmatrix}$	Bi-mode’<br>(2’)
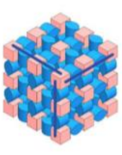	$\begin{bmatrix} 4.0e\text{-}2 & 1.0e\text{-}3 & 1.0e\text{-}3 & 0 & 0 & 0 \\ & 4.0e\text{-}2 & 1.0e\text{-}3 & 0 & 0 & 0 \\ & & 4.0e\text{-}2 & 0 & 0 & 0 \\ & & & 3.6e\text{-}5 & 0 & 0 \\ & sym & & & 3.6e\text{-}5 & 0 \\ & & & & & 3.6e\text{-}5 \end{bmatrix}$	**3.6e-5**<br>**3.6e-5**<br>**3.6e-5**<br>3.9e-2<br>3.9e-2<br>4.2e-2	$\begin{pmatrix}0\\0\\0\\1\\0\\0\end{pmatrix}, \begin{pmatrix}0\\0\\0\\0\\1\\0\end{pmatrix}, \begin{pmatrix}0\\0\\0\\0\\0\\1\end{pmatrix}$	Tri-mode<br>(3)
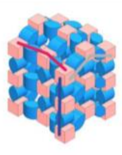	$\begin{bmatrix} 2.0e\text{-}2 & 0 & 7.6e\text{-}4 & 0 & 0 & 0 \\ & 2.9e\text{-}4 & 0 & 0 & 0 & 0 \\ & & 5.1e\text{-}2 & 0 & 0 & 0 \\ & & & 4.5e\text{-}5 & 0 & 0 \\ & sym & & & 4.1e\text{-}3 & 0 \\ & & & & & 4.9e\text{-}5 \end{bmatrix}$	**4.5e-5**<br>**4.9e-5**<br>**2.9e-4**<br>4.1e-3<br>2.0e-2<br>5.1e-2	$\begin{pmatrix}0\\0\\0\\1\\0\\0\end{pmatrix}, \begin{pmatrix}0\\0\\0\\0\\0\\1\end{pmatrix}, \begin{pmatrix}0\\1\\0\\0\\0\\0\end{pmatrix}$	Tri-mode’<br>(3’)
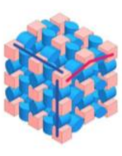	$\begin{bmatrix} 2.4e\text{-}4 & 0 & 0 & 0 & 0 & 0 \\ & 4.1e\text{-}2 & 1.1e\text{-}3 & 0 & 0 & 0 \\ & & 4.1e\text{-}2 & 0 & 0 & 0 \\ & & & 3.7e\text{-}5 & 0 & 0 \\ & sym & & & 4.1e\text{-}5 & 0 \\ & & & & & 3.7e\text{-}5 \end{bmatrix}$	**3.7e-5**<br>**3.7e-5**<br>**4.1e-5**<br>**2.4e-4**<br>4.0e-2<br>4.2e-2	$\begin{pmatrix}0\\0\\0\\1\\0\\0\end{pmatrix}, \begin{pmatrix}0\\0\\0\\0\\0\\1\end{pmatrix}, \begin{pmatrix}0\\0\\0\\0\\1\\0\end{pmatrix}, \begin{pmatrix}1\\0\\0\\0\\0\\0\end{pmatrix}$	Quadra-mode<br>(4)
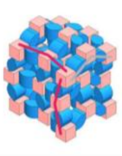	$\begin{bmatrix} 2.1e\text{-}2 & 0 & 0 & 0 & 0 & 0 \\ & 3.0e\text{-}4 & 0 & 0 & 0 & 0 \\ & & 3.0e\text{-}4 & 0 & 0 & 0 \\ & & & 5.1e\text{-}5 & 0 & 0 \\ & sym & & & 4.0e\text{-}3 & 0 \\ & & & & & 5.2e\text{-}5 \end{bmatrix}$	**5.1e-5**<br>**5.2e-5**<br>**3.0e-4**<br>**3.0e-4**<br>4.0e-3<br>2.1e-2	$\begin{pmatrix}0\\0\\0\\1\\0\\0\end{pmatrix}, \begin{pmatrix}0\\0\\0\\0\\0\\1\end{pmatrix}, \begin{pmatrix}0\\1\\0\\0\\0\\0\end{pmatrix}, \begin{pmatrix}0\\0\\1\\0\\0\\0\end{pmatrix}$	Quadra-mode’<br>(4’)
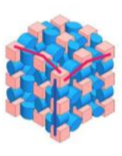	$\begin{bmatrix} 2.5e\text{-}4 & 0 & 0 & 0 & 0 & 0 \\ & 2.5e\text{-}4 & 0 & 0 & 0 & 0 \\ & & 4.3e\text{-}2 & 0 & 0 & 0 \\ & & & 3.9e\text{-}5 & 0 & 0 \\ & sym & & & 4.3e\text{-}5 & 0 \\ & & & & & 4.3e\text{-}5 \end{bmatrix}$	**3.9e-5**<br>**4.3e-5**<br>**4.3e-5**<br>**2.5e-4**<br>**2.5e-4**<br>4.3e-2	$\begin{pmatrix}0\\0\\0\\1\\0\\0\end{pmatrix}, \begin{pmatrix}0\\0\\0\\0\\0\\1\end{pmatrix}, \begin{pmatrix}0\\0\\0\\0\\1\\0\end{pmatrix}, \begin{pmatrix}1\\0\\0\\0\\0\\0\end{pmatrix}, \begin{pmatrix}0\\1\\0\\0\\0\\0\end{pmatrix}$	Penta-mode<br>(5)
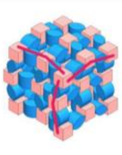	$\begin{bmatrix} 2.5e\text{-}4 & 0 & 0 & 0 & 0 & 0 \\ & 2.5e\text{-}4 & 0 & 0 & 0 & 0 \\ & & 2.5e\text{-}4 & 0 & 0 & 0 \\ & & & 4.3e\text{-}5 & 0 & 0 \\ & sym & & & 4.3e\text{-}5 & 0 \\ & & & & & 4.3e\text{-}5 \end{bmatrix}$	**4.3e-5**<br>**4.3e-5**<br>**4.3e-5**<br>**2.5e-4**<br>**2.5e-4**<br>**2.5e-4**	$\begin{pmatrix}0\\0\\0\\1\\0\\0\end{pmatrix}, \begin{pmatrix}0\\0\\0\\0\\1\\0\end{pmatrix}, \begin{pmatrix}0\\0\\0\\0\\0\\1\end{pmatrix}, \begin{pmatrix}1\\0\\0\\0\\0\\0\end{pmatrix}, \begin{pmatrix}0\\1\\0\\0\\0\\0\end{pmatrix}, \begin{pmatrix}0\\0\\1\\0\\0\\0\end{pmatrix}$	Hexa-mode<br>(6)

Supplementary Fig. 9 The normalized effective stiffness matrix, the calculated eigenvalues, the corresponding zero mode and the classification of each 3D metamaterial. All eigenvalues are normalized by the young’s modulus of the constituent E_0.

Supplementary Note 6. Effective compressive and shear moduli of the ten configurations

We first transform the printed tessellation sample from the penta-mode configuration to the hexa-mode configuration with the fixture C. Sequentially, quadra-mode, tri-mode, bi-mode, uni-mode, null-mode, bi-mode', and quadra-mode' configurations can be obtained with eight fixtures D, E, F, G, H, I and J, respectively. These eight fixtures and their corresponding transformed configurations are shown in Supplementary Fig. 10. Specifically, the penta-mode and tri-mode' configurations are obtained with fixtures A and B, as shown in Supplementary Fig. 6.

We also conduct compression and shear tests for each configuration and all ten tessellations' compressive and shear moduli are experimentally obtained. On the other hand, we can calculate the effective compressive (or tensile) moduli E_i and shear moduli G_i $(i = x, y, z)$ of each metamaterial configuration along the three orthogonal directions based on the compliance matrix $\mathbf{s}^{eff}$, which has the following relation [1]

$$\mathbf{s}^{eff} = (\mathbf{c}^{eff})^{-1} = \begin{bmatrix} 1/E_x & -v_{yx}/E_y & -v_{zx}/E_z & 0 & 0 & 0 \\ & 1/E_y & -v_{zy}/E_z & 0 & 0 & 0 \\ & & 1/E_z & 0 & 0 & 0 \\ & & & 1/G_y & 0 & 0 \\ & sym. & & & 1/G_z & 0 \\ & & & & & 1/G_x \end{bmatrix} \tag{7}$$

where $\mathbf{c}^{eff}$ is the effective stiffness matrix obtained with the homogenization method. Finally, the experimentally obtained moduli and numerically calculated moduli are normalized with the measured and calculated shear moduli of the tri-mode metamaterial, respectively.

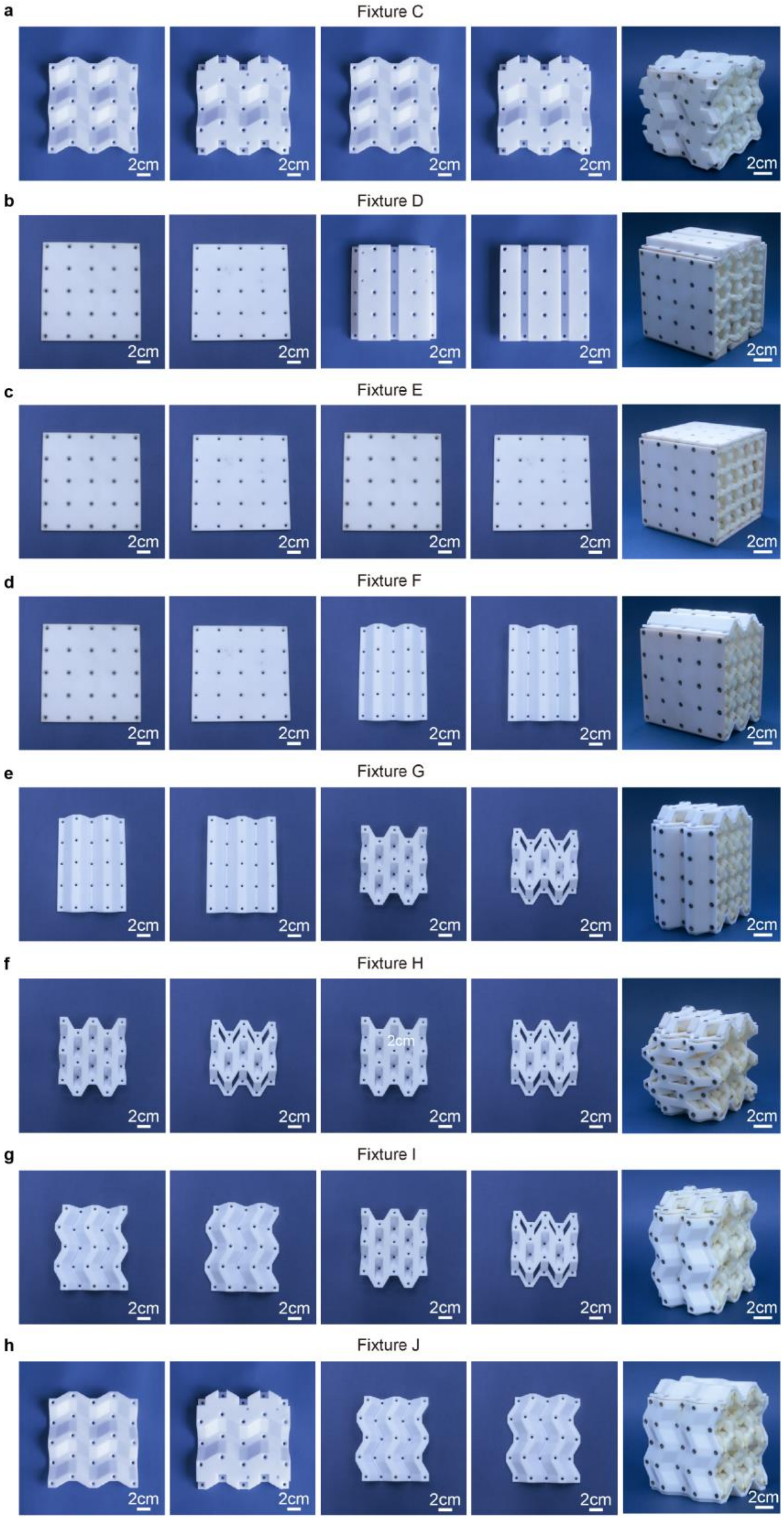

Supplementary Fig. 10 Photograph of fixtures used to reconfigure the sample. a. Fixture C for the hexa-mode configuration. **b.** Fixture D for the quadra-mode configuration. **c.** Fixture E for the tri-mode configuration. **d.** Fixture F for the bi-mode configuration. **e.** Fixture G for the uni-mode configuration. **f.** Fixture H for the null-mode configuration. **g.** Fixture I the for bi-mode' configuration. **h.** Fixture J for the quadra-mode' configuration.

Supplementary Note 7. Effects of different geometrical parameters

In this part, we first investigate the effects of folding angles on the normalized effective moduli along three orthogonal directions to validate the decoupled orthogonal mechanism design. In Supplementary Fig. 11, one can found that the elastic modulus changes only when the folding angle along the same direction changes, which validates the decoupled design.

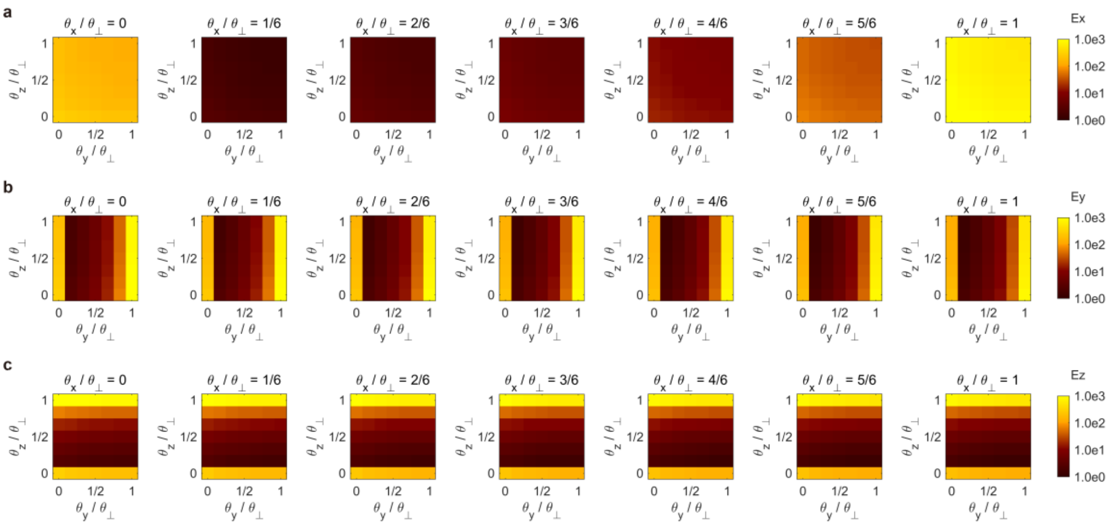

Supplementary Fig. 11 The effects of three folding angles ($\theta_i, i = x, y, z$) on three normalized effective compression moduli. a Ex. **b** Ey. **c** Ez. $\theta_\perp$ is defined as the maximum value of the folding angle. The thickness of the hinge $h_0 = 1$ mm and the length of the rhombus $L_0 = 10$ mm. all moduli are normalized by the minimal one.

Second, we conduct calculations of Force-Displacement curves based on the FE model. Different geometrical properties, including the folding angle θ_z (θ_x and θ_y have the same effect due to the decoupled orthogonal design), the length of diamond bars L_0, and the thickness of the hinge h_0, are chosen. The results are shown in Supplementary Fig. 12. It can be found that changing the folding angle can result in qualitative transformation of the metamaterial, while changing the other two geometrical properties can only affect the elastic moduli quantitatively. To be specific, when $\theta_z/\theta_\perp = 1$, the metamaterial is in CE configurations along the three orthogonal directions and therefore, it is a tri-mode with three shear zero modes. When $\theta_z/\theta_\perp$ decreases ($\theta_z/\theta_\perp \neq 0$), the metamaterial is in PF configuration along z direction and therefore, the metamaterial's compressive deformation along the z direction becomes a

zero mode. When $\theta_z/\theta_\perp = 0$, the metamaterial is in CF configuration along z direction and therefore, two zero modes (the compressive deformation along the z direction and the shear deformation in the z-y plane) disappear, as shown in Supplementary Fig. 12a. When we change the length of diamond bars L_0 and the thickness of the hinge h_0 ($\theta_z/\theta_\perp = \theta_x/\theta_\perp = \theta_y/\theta_\perp = 1$ being fixed), the tri-mode state (with only three shear zero modes) stays unchanged, as shown in Supplementary Fig. 12b and 12c, respectively. It can be concluded that the number of zero mode depends majorly on the folding angle, while both the thickness of the hinge h_0 and the length of the rhombus L_0 have little effect on the zero mode.

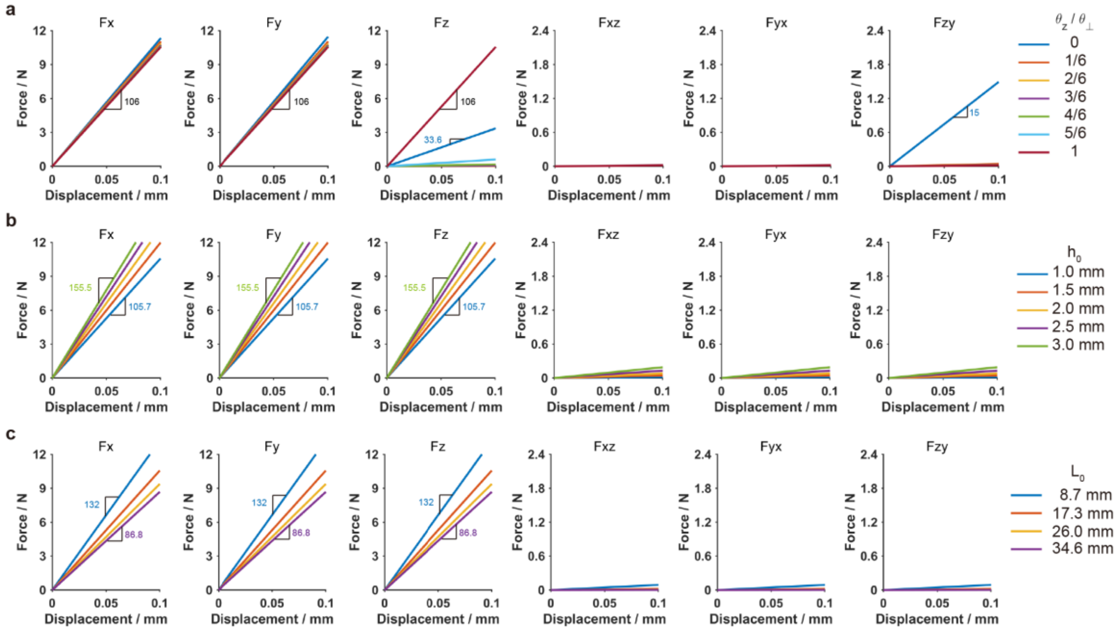

Supplementary Fig. 12 Predicted Force-Displacement curves of metamaterials with different geometrical parameters using a FE approach. a The effect of the folding angle θ_z on the Force-Displacement curves, where $\theta_x/\theta_\perp = \theta_y/\theta_\perp = 1, L_0 = 10$ mm, $h_0 = 1$ mm. **b** The effect of the thickness of the hinge h_0 on the Force-Displacement curves, where $\theta_x/\theta_\perp = \theta_y/\theta_\perp = \theta_z/\theta_\perp = 1, L_0 = 10$ mm. **c** The effect of the length of the rhombus L_0 on the Force-Displacement curves, where $\theta_x/\theta_\perp = \theta_y/\theta_\perp = \theta_z/\theta_\perp = \theta_\perp, h = 1$ mm.

Supplementary Note 8. Dynamic experiments on the transformable metamaterial

To illustrate the unconventional dynamic properties of the transformable metamaterial, we conduct an experiment for 1D polarized elastic wave manipulation with the fabricated transformable metamaterial sample. A 6×6 sample is fabricated for

the dynamic experiment. The sample is made of TPU (E_{TPU} = 48.9 MPa, υ_{TPU} = 0.4, ρ_{TPU} = 1200 kg m^{-3}) and is in CE configuration with in-plane translational lattice constant being 29 mm (corresponding to $\lambda_{\mathrm{L}}/24$, where λ_{L} represents the wavelength of longitudinal (L) wave in the metamaterial at 80 Hz) and with out-of-plane thickness being 5 mm. Considering the 3D printing accuracy, the smallest size for the compliant joints is 0.6 mm.

The experimental setup is shown in Supplementary Fig. 13. One end of the sample is fixed on a metal fixture, while the other ends are free. The metal fixture is constrained on a slide track so that it can only move along one direction. A harmonic wave (80 Hz) signal is generated by the scanning laser Doppler vibrometer (LDV) (Polytec PSV-500) and amplified with a wideband power amplifier (Krohn-Hite 7602M), which then drives a shaker (LDS V201). The harmonic wave excitation from the shaker is transformed to the left end of the sample via a stinger connected to the metal fixture. L or transverse (T) wave incidence can be generated by switching the connections between the stinger and the metal fixture. With the x-y coordinate being fixed to the sample, the excited L or T wave propagates along the x direction in the sample. The in-plane displacements (Ux and Uy) on the other end of the sample are measured via the LDV. All results are obtained by averaging five measurements and are normalized by the corresponding displacements of the metal fixture.

Numerical harmonic wave simulations with the FE model (with exact geometry) of the sample are also conducted. First, same boundary conditions as in the experiment are applied to the FE sample. Next, two displacement probes are defined on the left and right sides (same positions as the experiment) to obtain the displacement amplitudes. Finally, normalized displacements (the ratios of displacement amplitudes on the right side to that on the left side) are obtained and compared with those obtained from the experimental measurements, as shown in the second column of Fig. 4a in the main text.

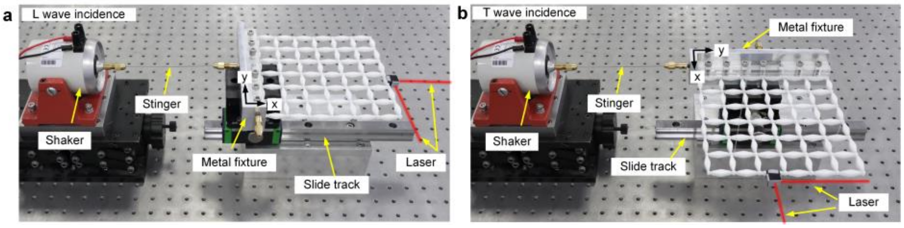

Supplementary Fig. 13 Dynamic experiment setup for L (a) and T (b) wave incidences.

Furthermore, we conduct same numerical harmonic wave simulations with homogenized effective metamaterial models. Since the effective uni-mode metamaterial has a negligible shear modulus but a substantial effective longitudinal modulus, so the incident L wave is totally transmitted (Ux = 1 and Uy = 0), while the incident T wave are completed blocked (Ux = 0 and Uy = 0). For effective bi-mode and null-mode metamaterials, numerical harmonic wave simulations are conducted accordingly. Experimental measurement results and numerical simulation results on FE models as well as on the homogenized effective metamaterial models are compared in bar chats (the third column of Fig. 4a in the main text).

Finally, to illustrate transmitted wave patterns, numerical wave simulations are also conducted on the length-extended FE metamaterial sample (with exact geometry) as well as the equivalent effective metamaterial sample. Absorbing boundary is applied to the right end and PBCs are applied to the upper and bottom sides to eliminate any unwanted wave reflection and the harmonic L or T wave is excited on the left end, as shown in Supplementary Fig. 14. For the FE model, it consists of 84 unit cells, and each unit cell is discretized into about 1500 triangular elements. The wavelength of the incident L wave is 15 times larger than the size of a unit cell. The equivalent effective model has the same size and boundary conditions as the FE model, and the maximum mesh size is controlled to be one tenth of the wavelength. The simulation results are shown in the fourth column of Fig. 4a in the main text.

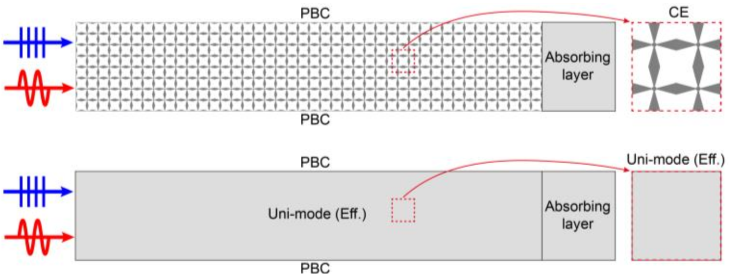

Supplementary Fig. 14 The length-extended models of the FE sample and the equivalent effective metamaterial sample for the uni-mode (CE).

By using the similar reconfiguration process in the static experiments, we transform the sample's CE configuration into the PF-x configuration (bi-mode) with the fixture K, then into the CF configuration (null-mode) with the fixture L, as shown in Supplementary Fig. 15. Both fixtures are fabricated via 3D printed nylon materials. Similar dynamics experiments are performed on the sample after each reconfiguration, and the results are compared with the numerical wave simulations on FE and effective models.

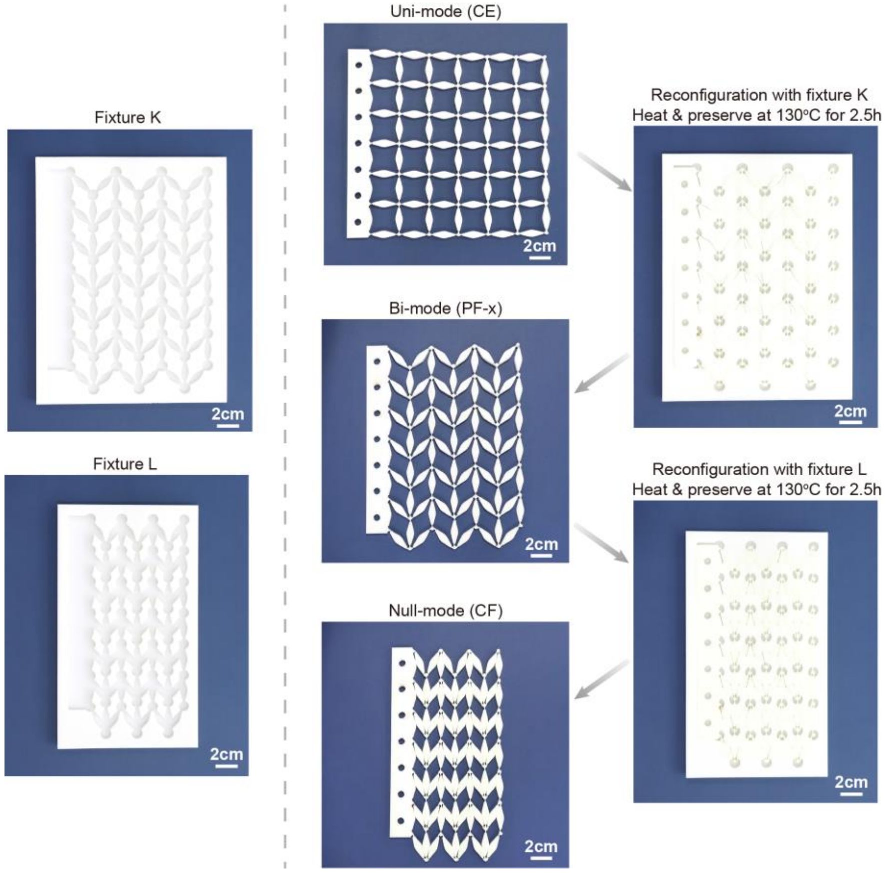

Supplementary Fig. 15 Reconfiguration paths of the sample for dynamic experiments.

Since the metamaterial's ability of polarized elastic wave manipulation is based on its reconfigurable effective elasticity, we demonstrate its frequency insensitivity by choosing different harmonic wave frequencies, as shown in Supplementary Fig. 16.

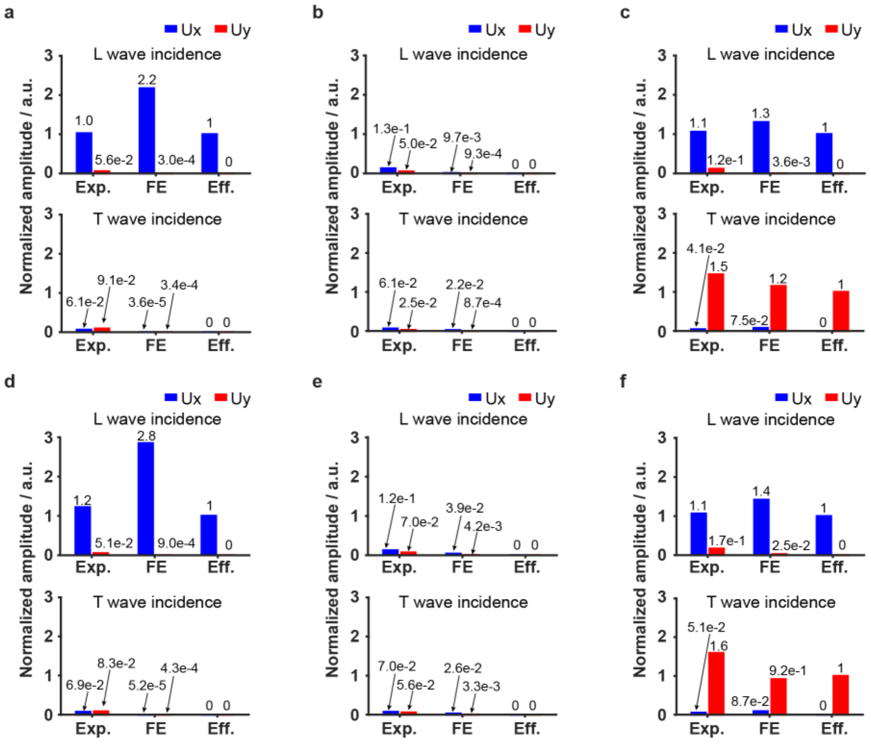

Supplementary Fig. 16 Frequency insensitivity of polarized elastic wave manipulation via the transformable metamaterial sample. Measured (Exp.) results, simulated results with FE model and effective (Eff.) model, **a-c** at 90 Hz, **d-f** at 100 Hz.

Supplementary Note 9. 2D and 3D wave analysis of transformable metamaterials

Here, we provide detailed analysis on the wave behaviors of the 2D and 3D transformable metamaterials based on the FE models and the equivalent effective metamaterial models.

For wave analysis in 2D metamaterials, the microstructures and the effective elastic stiffnesses of the uni-mode and bi-mode metamaterials shown in Supplementary Table 1 are selected, and effective mass densities are shown in Supplementary Table 3. FE models used for the simulations consist of more than 2000 unit cells and have at least 18 unit cells along the x or y direction. Absorbing layers are adopted on the right and upper ends of the FE models to avoid unnecessary reflected waves. A Gaussian beam

of the L wave with operating frequency 10 kHz is launched along 45° direction from the left-bottom end of the FE sample. Same Gaussian beam and absorbing layers are applied to the equivalent effective metamaterial models. Harmonic wave simulations on the FE models as well as the equivalent effective metamaterial models are performed with COMSOL Multiphysics. In the uni-mode metamaterial (with all CE configurations), same 90° wave-splitting results can be found in both FE and equivalent effective models, which confirms the accuracy of the equivalent effective model. In the bi-mode metamaterial (with the PF-y configuration), x-directional wave propagation can be observed in the wave simulation results of both models. However, another wave with much shorter wavelength propagating about 50 degrees away from the primary x-directional wave can be found in the result of the FE model, but absence in the result of equivalent effective model. This discrepancy comes from the weakened (not ideal) linkage design in the FE model.

	2D		3D	
	Uni-mode	Bi-mode	Tri-mode	Penta-mode
ρ_{eff}/ρ_0	2.7e-1	3.9e-1	2.5e-1	2.7e-1

Supplementary Table 3 The normalized effective mass density. ρ_0 represents the mass density of the constituent material.

To better understand the unique wave direction manipulations in the 2D and 3D metamaterials, the wave propagation characteristics in the effective homogenous media are investigated. Plane harmonic elastic wave propagations are determined by the Christoffel equation [9]:

$$\left(\vec{\mathbf{k}} \cdot \mathbf{c}^{eff} \cdot \vec{\mathbf{k}}\right)\vec{v} = \rho_{eff}\omega^2\vec{\mathbf{v}} \tag{8}$$

where $\vec{\mathbf{k}}$ is the wave vector, $\vec{\mathbf{v}}$ is the wave polarization velocity vector, and ω is the angular frequency. Moreover, ρ_{eff} and $\mathbf{c}^{eff}$ represent the effective mass density and elastic tensor of a metamaterial, respectively. Therefore, an eigenvalue problem forms and its solution determines the dispersive nature of the metamaterial. By specifying a frequency, equi-frequency curves (EFCs) of uni-mode and bi-mode metamaterials can

be drawn by calculating components of wave vectors along all directions.

EFCs of the uni-mode metamaterial are shown in Supplementary Fig. 17a. Red arrows normal to the EFCs indicate the wave energy propagation directions. First, when a wave propagates along the x or y direction, only one type of wave can be identified with its energy propagation direction being along the x or y direction. Second, when the propagation direction is away from the x or y direction, two types of waves can be identified with their energy propagation directions being always parallel with the x and y directions, respectively. Such strong anisotropic wave behavior in the uni-mode metamaterial explains the unique 90º wave-splitting results for the 45º L wave beam incidence in Fig. 4b in the main text.

EFCs of the bi-mode metamaterial with PF-y configuration are shown in Supplementary Fig. 17b. First, no wave propagates along the y direction. Second, when the propagation direction is away from the y direction, only one wave can be identified with its energy propagation direction being always parallel with the x direction, which also explains the only x-directional wave propagation result in Fig. 4c in the main text.

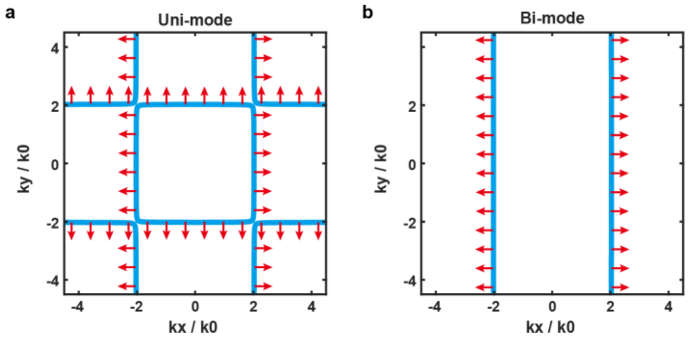

Supplementary Fig. 17 Equi-frequency curves (EFCs) of the 2D transformable metamaterial. a. The EFCs of the uni-mode metamaterial. **b.** The EFC of the bi-mode metamaterial. kx and ky represent two components of the wave vector, and k0 defined as $k0 = \omega/\sqrt{E_0/\rho_0}$, represents the wavenumber of the constituent material.

For wave analysis in 3D metamaterial, equivalent effective models of tri-mode (3) and penta-mode (5) are investigated. Each model is obtained by cutting a corner of a cube along the orange triangle area normal to the body diagonal of the cube (as illustrated in Fig. 4d-e in the main text). L wave incidence with a Gaussian distribution

on the blue circle area is applied, and the operating frequency is 10 kHz. Absorbing layers are also applied to all three square ends to eliminate boundary reflections. Since the 3D tri-mode metamaterial is the 3D counterpart of the 2D uni-mode metamaterial, a three-wave-splitting phenomenon can be observed in Fig. 4d in the main text. On the other hand, the 3D penta-mode metamaterial (with CE-z, PF-x, and PF-y configuration) is the 3D counterpart of the 2D bi-mode metamaterial and therefore, the incident L wave only propagates along the z direction, as shown in Fig. 4e in the main text.

Supplementary References

[1] Nemat-Nasser, S., Hori M., Micromechanics: Overall Properties of Heterogeneous Materials (Elsevier, 2013).

[2] Phani, A. Srikantha, Hussein, Mahmoud I. Dynamic of Lattice Materials (John Wiley & Sons, 2017).

[3] Liu, X. N., Hu, G. K., Huang, G. L., Sun, C. T. An elastic metamaterial with simultaneously negative mass density and bulk modulus. *Appl. Phys. Lett.* **98**, 251907 (2011).

[4] Hutchinson, R.G., Fleck, N.A. The structural performance of the periodic truss. *J. Mech. Phys. Solids* **54**, 756–782 (2006).

[5] Wang, K., Cai, M., Zhou, P. Z. Hu, G. K. Homogenization in a simpler way: analysis and optimization of periodic unit cells with Cauchy–Born hypothesis. *Struct. Multidiscip. Optim.* **64,** 3911-3935 (2021).

[6] Auld, B. A., Acoustic fields and waves in solids. (John Wiley & Sons, 1973).

[7] Milton, G. W., Cherkaev A. V., *J. Eng. Mater. Technol.*, **117**, 483 (1995).

[8] Sun, C. T., Vaidya R. S., *Compos. Sci. Technol.*, **56**, 171 (1996).

[9] Brillouin, L., Wave Propagation in Periodic Structures (Dover, New York, 1953).
