# Supplementary material for: Engineering zero modes in transformable mechanical metamaterials
Source: Nat Commun. 2023 Mar 7;14:1266. doi: 10.1038/s41467-023-36975-2 (PMC9992356; doi:10.1038/s41467-023-36975-2)
Supplement: Supplementary file 2 — Description of Additional Supplementary Files [file 41467_2023_36975_MOESM2_ESM.pdf]

Description of Additional Supplementary Files

File Name: Supplementary Movie 1
Description: The reconfiguration of the 2D tessellation.

File Name: Supplementary Movie 2
Description: Decoupled motion of 3D unit.

File Name: Supplementary Movie 3
Description: Reconfiguration and experimental characterization of the transformable metamaterial.

File Name: Supplementary Movie 4
Description: Transformation process of the 3D metamaterial unit cell with 10 configurations.

File Name: Supplementary Movie 5
Description: Transformability validation of ten configurations via reconfiguration experiments.

File Name: Supplementary Movie 6
Description: 2D wave functions in the FE metamaterial sample.
